# TOPK Inhibition Promotes Anti‐Tumor Immunity Via eIF4F Complex Mediated STAT1 Translation in Gastric Cancer

**DOI:** 10.1002/advs.202517380

**Published:** 2025-12-19

**Authors:** Junbing Chen, Longtao Huangfu, Gangjian Wang, Xueying Wang, Huanbo Zhu, Qian Yao, Cong Chen, Xiaohuan Tang, Ting Guo, Biao Fan, Xingyang Liu, Qingda Li, Zining Liu, Ying Hu, Tianze Sun, Jiafu Ji, Xiaofang Xing

**Affiliations:** ^1^ Key Laboratory of Carcinogenesis and Translational Research (Ministry of Education) Division of Gastrointestinal Cancer Translational Research Laboratory Peking University Cancer Hospital and Institute Beijing 100142 China; ^2^ Department of Hepatobiliary Surgery & Liver Transplantation Liver Cancer Institute Zhongshan Hospital Fudan University Key Laboratory of Carcinogenesis and Cancer Invasion Ministry of Education Shanghai 200032 China; ^3^ Department of Gastrointestinal Surgery Peking University Cancer Hospital and Institute Beijing 100142 China; ^4^ Program in MPE Molecular Pathological Epidemiology Department of Pathology Brigham and Women's Hospital Harvard Medical School Boston MA 02215 USA; ^5^ Key Laboratory of Carcinogenesis and Translational Research (Ministry of Education/Beijing) Laboratory of Molecular Oncology Peking University Cancer Hospital & Institute Beijing 100142 China; ^6^ Department of Gynecologic Oncology National Cancer Center/National Clinical Research Center for Cancer/Cancer Hospital Chinese Academy of Medical Sciences & Peking Union Medical College Beijing 100021 China; ^7^ Biological Sample Bank Peking University Cancer Hospital & Institute Fu‐Cheng Road Beijing 100142 China; ^8^ State Key Laboratory of Holistic Integrative Management of Gastrointestinal Cancers Beijing Key Laboratory of Carcinogenesis and Translational Research Division of Gastrointestinal Cancer Translational Research Laboratory Peking University Cancer Hospital & Institute Beijing 100142 China

**Keywords:** gastric cancer, immunometabolic microenvironment, immunotherapy, PD‐L1, TOPK

## Abstract

Immune checkpoint blockade‐directed immunotherapy emerges as a revolutionary therapy in gastric cancer (GC). However, the proportion of patients who can benefit from it and its overall efficacy remain limited. Here the aim is to identify key dual‐function targets that both inhibit proliferation and suppress immune evasion. Using whole genome‐wide CRISPR‐Cas9‐based screening, the serine/threonine kinase T‐lymphokine‐activated killer cell‐originated protein kinase (TOPK) is identified as a key regulator of PD‐L1 in gastric cancer upon IFN‐γ stimulation. Mechanical study is performed to explore the role of TOPK in promoting GC malignancy and immune evasion in vitro and vivo. Higher TOPK levels in tumor tissues are observed, correlated with clinical stages, efficacy and survival. Upon IFN‐γ stimulation, TOPK phosphorylates eIF4F complex component eIF4A1 to increase its unwinding activity of STAT1 mRNA, enhancing STAT1 translation efficiency. This process leads to adaptive overexpression of PD‐L1 and IDO1, resulting in immunometabolic suppression through PD‐L1‐mediated inhibition, IDO1‐induced tryptophan depletion and kynurenine production. TOPK inhibitors reshape tumor immunometabolic microenvironment to trigger anti‐tumor immunity in GC. The IFN‐γ‐TOPK‐eIF4F‐STAT1‐PD‐L1/IDO1 axis as a crucial regulator of the tumor immunometabolic microenvironment and provide novel insights into the combination of targeted therapy and immunotherapy for GC treatment.

## Graphical abstract

This study identifies TOPK as a dual‐function target in gastric cancer: it drives tumor cell proliferation and migration, and under IFN‐γ stimulation, phosphorylates eIF4F complex component eIF4A1 to enhance STAT1 mRNA translation, thereby promoting PD‐L1/IDO1‐mediated tumor immunometabolic evasion. Notably, TOPK inhibitors reshape the immunometabolic microenvironment and synergize with immunotherapy, providing a novel therapeutic strategy.

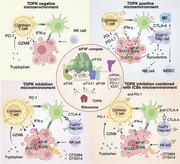

## 1 Introduction

Gastric cancer (GC) ranks among the five most common malignancies worldwide in terms of both incidence and mortality rates. Targeted therapy, immunotherapy, and combined therapy are emerging as new options. Two main molecular biomarkers, HER2 and PD‐L1, have been validated to guide precise treatment in GC.^[^ ^1^ ^]^ Despite emerging targets like FGFR2, Claudin‐18.2, their coverage is limited (e.g., 12–23% of patients with HER2 positivity), and the efficacy of related treatment options remains limited. However, outcomes for patients with GC receiving immunotherapy alone or with chemotherapy remain unsatisfactory. To enhance immunotherapy effectiveness and suppress GC's rapid proliferation and immune evasion, we aimed to identify key dual‐function targets that promote proliferation and mediate immune resistance.

T‐lymphokine‐activated killer cell‐originated protein kinase (TOPK), also known as PDZ‐binding kinase (PBK), is a serine/threonine protein kinase implicated in cell cycle regulation and apoptosis resistance. Many cancers, including GC, display high TOPK expression, promoting tumor proliferation, invasion, metastasis, and survival.^[^ ^2^, ^3^ ^]^ Small molecules like OTS964 and OTS514 specifically antagonize TOPK, exhibiting significant inhibitory effects on tumor growth.^[^ ^4^, ^5^ ^]^ More importantly, TOPK is correlated with immune cells infiltration in the tumor microenvironment,^[^ ^6^, ^7^, ^8^, ^9^, ^10^ ^]^ though the mechanism and therapeutic value remain unclear. JAK2, a key mediator of the IFN‐γ upstream pathway, can phosphorylate and activate TOPK, promoting Burkitt Lymphoma progression,^[^ ^11^ ^]^ suggesting a potential connection between TOPK and IFN‐γ stimulation.

IFN‐γ, a key cytokine in the tumor microenvironment secreted by cytotoxic immune cells,^[^ ^12^ ^]^ plays an important role in eliminating tumor cells in many cancers. It was thought to play a dual role in GC, inhibiting or enhancing tumor cells.^[^ ^13^ ^]^ GC cells, under IFN‐γ stimuli, can acquire adaptive immune resistance, suppressing immune cell cytotoxicity and promoting proliferation and metastasis.^[^ ^14^ ^]^ The mechanism of IFN‐γ‐induced immune checkpoints, including PD‐L1, is crucial in immune surveillance evasion. eIF4F‐mediated STAT1 translation efficiency (TE) can increase PD‐L1 surface levels under IFN‐γ stimuli, a process blocked by eIF4A1 intervention.^[^ ^15^ ^]^ Generally, eIF4F complex components can be activated under IFN‐γ conditions, playing a crucial role in tumor immune evasion by controlling immune checkpoints' translation.^[^ ^16^, ^17^ ^]^

The CRISPR‐Cas9 library‐screening approach offers comprehensive experimental results for elucidating gene functions through large‐scale phenotype selections. Consequently, key drivers that regulate PD‐L1 expression and immunity, including CMTM6,^[^ ^18^ ^]^ CTCF,^[^ ^19^ ^]^ IRF4, and BATF3, have been identified.^[^ ^20^ ^]^ Our previous research, utilizing CRISPR‐Cas9 genome‐wide screening without IFN‐γ stimuli, identified TRIM28 as regulating PD‐L1 in transcriptional and post‐translational pathways and inducing immune escape in GC. This discovery provided novel targets and perspectives for sensitizing treatment in GC.^[^ ^21^ ^]^ Here, based on CRISPR‐Cas9 screening with IFN‐γ stimuli, we further explored TOPK as the key druggable target mediating PD‐L1 and IDO1 overexpression and adaptive resistance in IFN‐γ stimuli in GC. We validated the molecular functions and biological roles of the TOPK‐eIF4A1‐STAT1 axis in regulating PD‐L1/IDO1, and reshaping the immunometabolic microenvironment and immune evasion. Finally, we assessed the potential effect of TOPK inhibitors.

## 2 Results

### 2.1 Integrated CRISPR and Compounds Screening Identifies Inhibitors Blocking IFN‐γ‐Induced PD‐L1

**Figure 1:**
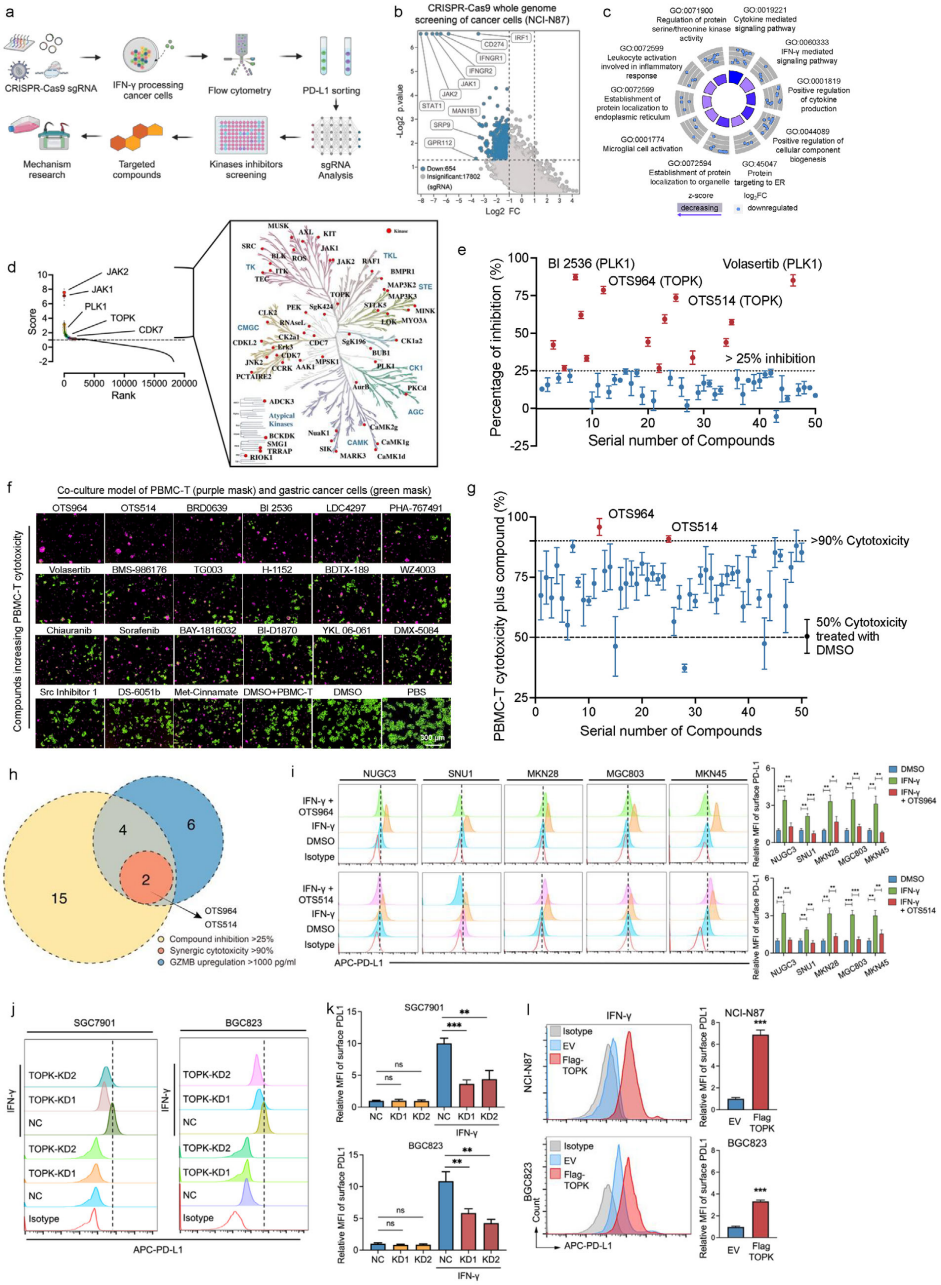
Integrated CRISPR and compounds screening identifies inhibitors blocking IFN‐γ‐induced PD‐L1. a) Diagram of the high‐throughput screening process combining CRISPR‐Cas9 whole‐genome editing with compound screening in cancer cells. b) CRISPR/Cas9 whole genome screening of targets mediating IFN‐γ induced PD‐L1 (CD274) overexpression in gastric cancer cells (NCI‐N87) based on MAGeCK‐VISPR algorithm. c) Gene Ontology (GO) pathway annotation of the potential mediators in IFN‐γ induced PD‐L1 pathway in gastric cancer cells. d) The sgRNA score and rank of targets mediating IFN‐γ induced PD‐L1 expression. The top 50 kinases potentially mediating the IFN‐γ/PD‐L1 pathway in gastric cancer cells were annotated in the human protein kinome dendrogram. e) The inhibition of the top 50 kinase inhibitors against gastric cancer cells for 48 h. Inhibitors with > 25% inhibition were selected for this study. f) Co‐culture model of PBMC‐T with gastric cancer cells in Incucyte‐assessed compounds showing increased T cell cytotoxicity against gastric cancer cells. g) Percentage of T cells cytotoxicity inhibition under conditions of inhibitor and co‐culture with gastric cancer cells for 48 h. Inhibitors with cytotoxicity >75% were selected for this study. h) Venn diagram intersection (OTS964, OTS514, BI 2536) of compound screening results. i) OTS964 and OTS514 decreases IFN‐γ induced membrane PD‐L1 protein in gastric cancer cells (NUGC3, SNU1, MKN28, MGC803, MKN45). j,k) TOPK knockdown inhibits membrane PD‐L1 overexpression under IFN‐γ condition. The dashed line represents the median fluorescence intensity (MFI) of the negative control (NC) with IFN‐γ group. l) TOPK overexpression (EV) promotes membrane PD‐L1 overexpression under IFN‐γ conditions. Experiments were independently performed at least three times in vitro. Error bars represent the mean±SD of independent replicates. *p*‐values were determined using unpaired two‐tailed Student's *t*‐tests. ^*^ *p* < 0.05, ^**^ *p* < 0.01, ^***^ *p* < 0.001.

It was reported that IFN‐γ promotes cancer cell immune evasion,^[^ ^22^ ^]^ and PD‐L1 and IDO1 were the important IFN‐γ‐downstream immune checkpoints in many cancers.^[^ ^23^, ^24^, ^25^, ^26^ ^]^ To identify potential targets mediating IFN‐γ induced immune evasion in gastric cancer, the study first focuses on IFN‐γ induced adaptive overexpression of PD‐L1, and CRISPR/Cas9‐based whole genome editing and compound screening was performed in IFN‐γ stimulated GC cells (**Figure** **1**a). The results showed the significance of STAT1 in IFN‐γ induced PD‐L1 expression in NCI‐N87 cells (Figure 1b). Furthermore, Gene Ontology (GO) and Kyoto Encyclopedia of Genes and Genomes (KEGG) pathway annotation were performed with the top potential genes regulating IFN‐γ‐induced PD‐L1 expression. Several important signaling pathways, including “Regulation of protein serine/threonine kinase activity,” “Protein targeting to endoplasmic reticulum” (Figure 1c), and “JAK‐STAT signaling pathway” (Figure S1a, Supporting Information), were enriched. Considering the crucial roles and target potential of kinases in the proliferation, metastasis,^[^ ^27^ ^]^ we focused on targeting the key kinases affecting PD‐L1 expression upon IFN‐γ stimulation. The top 50 protein kinases that exhibited significant differences in influencing PD‐L1 expression in GC cells were identified and depicted in the kinome dendrogram (Figure 1d), along with 50 known kinase inhibitors targeting them (Figure S1b, Supporting Information). In a real‐time monitoring model for labeled live cells in vitro (Figure S1c, Supporting Information), BI2536, Volasertib, OTS964, and OTS514 significantly inhibited GC cells proliferation (Figure 1e). BI2536, OTS964, and BMS‐935177 significantly increased the level of Granzyme B (GZMB) in the supernatant of the co‐culture with T cells from PBMC‐T (Figure S1d,e, Supporting Information). Moreover, OTS964, OTS514, BI2536, and BRD0639, significantly enhanced PBMC‐T cytotoxicity against GC cells (Figure 1f,g). Finally, OTS964, OTS514, and BI2536 exhibited significant inhibitory effects on GC cells, enhancement of supernatant GZMB level, and PBMC‐T cytotoxicity (Figure 1h; Table S1, Supporting Information).

Consistent with our molecular docking analysis, both OTS964 and OTS514 were found to stably bind to the active pocket of TOPK, inhibiting its kinase activity and function (Figure S1f, Supporting Information). Furthermore, OTS964 and OTS514 was validated to block IFN‐γ‐induced elevated membrane PD‐L1 in GC cell lines (NUGC3, SNU1, MKN28, MGC803, MKN45) using flow cytometry (Figure 1i). Based on the different TOPK expression levels (Figure S1g, Supporting Information), we generated cells with stable TOPK overexpression (Flag‐TOPK) or depletion (TOPK‐KD) (Figure S1h, Supporting Information). *TOPK* knockdown decreased while its enforcing expression increased (Figure 1j–l).

### 2.2 Protein Kinase TOPK Inhibitors Efficiently Suppresses Gastric Cancer Proliferation

To investigate the potential effect of TOPK inhibitors (OTS964 and OTS514) on GC cells, a real‐time monitoring model assay was performed, revealing that these inhibitors markedly suppressed cancer cells proliferation in a dose‐ and time‐dependent manner (Figure S2a,b, Supporting Information). OTS964 inhibited GC cell lines (SGC7901, BGC823, and NCI‐N87) proliferation in colony‐forming unit (CFU) assays (Figure S2c,d), accompanied by increased apoptosis (Figure S2e, Supporting Information). Notably, OTS964 readily arrested GC cells in the G2/M phase (Figure S2f, Supporting Information) and disrupted mitochondrial membrane potential, as measured by JC‐1 staining (Figure S2g, Supporting Information). Furthermore, OTS964 and OTS514 decreased the size and 3D viability of tumor spheroids (Figure S2h, Supporting Information). Lastly, OTS964 administration significantly repressed BGC823‐derived percutaneous tumor growth in BALB/c nude mice without body weight loss (Figure S2I, Supporting Information).

### 2.3 High Expression of Protein Kinase TOPK Promotes Proliferation, Invasion, and Metastasis of Gastric Cancer

Initial investigations revealed that TOPK was universally expressed in various cancers in the Cancer Genome Atlas (TCGA) database, with significantly higher expression in tumors compared to normal tissues in the Stomach Adenocarcinoma (STAD) subset (Figure S3a, Supporting Information). Similarly, tumors from the Asian Cancer Research Group (ACRG) and Peking University Cancer Hospital (PUCH) had significantly higher TOPK expression in comparison to normal tissues (Figure S3b, Supporting Information). In the STAD data (*n* = 188), TOPK‐related pathways using GO and KEGG analyses were enriched in “ribonucleoprotein complex biogenesis,” “cell cycle G2/M phase transition', ‘RNA localization” (Figure S3c, Supporting Information). TOPK was highly expressed in epithelial cells with a high proliferative capacity, as evidenced by high KI67 expression in GC tumor tissues (Figure S3d, Supporting Information), indicating TOPK's malignant potential in GC. Spearman correlation analysis revealed a significant positive association between TOPK expression and pathways like “DNA replication,” “G2M checkpoint” (Figure S3e, Supporting Information). Furthermore, proteomics and phosphoproteomics analyses revealed that *TOPK* depletion could decrease pathways of “regulation of cell migration,” ‘cell growth’ (Figure S3f, Supporting Information). Phospho‐proteins enriched pathways included “mRNA 5′‐UTR binding', ‘translation activator activity” (Figure S3g, Supporting Information), suggesting a potential role for TOPK in regulating RNA translation. Serine/threonine motifs analysis revealed TOPK's preference for serine in TOPK‐regulated substrates (Figure S3h, Supporting Information). The protein domain enrichment analysis of differentially expressed proteins highlighted structural domains potentially involved in the phosphorylated substrates of TOPK (Figure S3i, Supporting Information).

TOPK expression modulates GC cells proliferation in real‐time Incucyte model (Figure S4a, Supporting Information). These findings were validated using 3D tumor spheroids in the real‐time high content imaging (HCI) monitoring model (Figure S4b,c, Supporting Information). Similar results were observed with EdU staining, while opposite results were observed in apoptosis‐associated terminal deoxynucleotidyl transferase dUTP nick‐end labeling (TUNEL) assays (Figure S4d, Supporting Information). *TOPK*‐depletion decreased, whereas its expression enhanced GC colony formation, as measured by CFU assays (Figure S4e, Supporting Information). It was observed for the positive regulation of GC cells migration and invasion by TOPK, as detected by scratch healing and Transwell assays in vitro (Figure S4f,g, Supporting Information). In vivo, the cell‐derived xenograft (CDX) model demonstrated that *TOPK* depletion inhibited percutaneous tumor growth, while *TOPK* overexpression promoted tumor growth (Figure S4h, Supporting Information). In mouse lung metastasis models of gastric cancer, *TOPK* depletion inhibited metastasis and tumor growth (Figure S4I, Supporting Information).

### 2.4 TOPK Mediates IFN‐γ Induced PD‐L1 and IDO1 Expression

**Figure 2:**
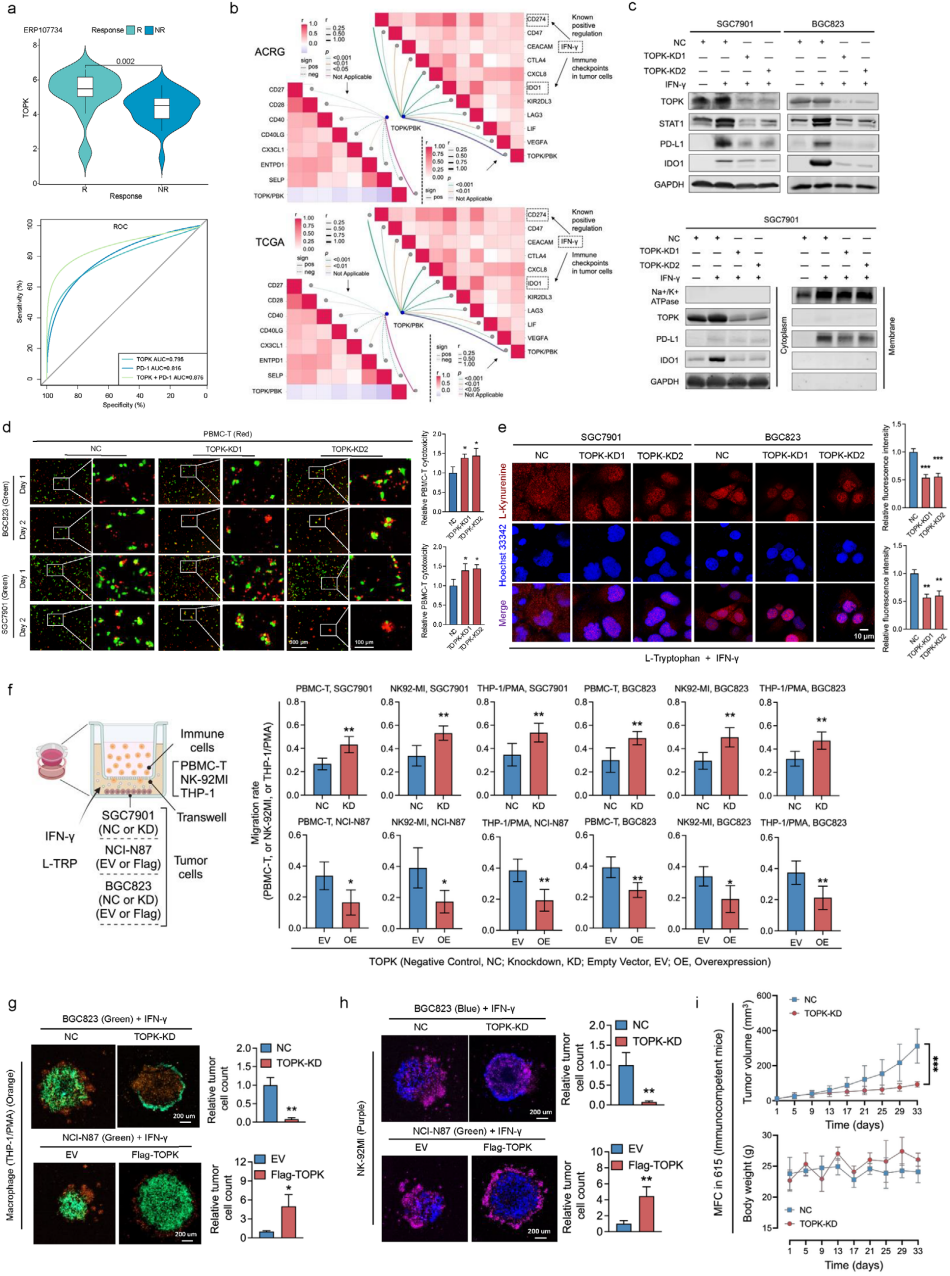
TOPK mediates IFN‐γ induced PD‐L1 and IDO1 expression in gastric cancer. a) Patients with better efficacy of immune checkpoint therapy had higher TOPK expression in the ERP107734 database. A combined panel of TOPK and PD‐1 expression demonstrated an elevated area under the curve (AUC) for predicting the efficacy of anti‐PD‐1 therapy. b) In ACRG and TCGA‐STAD databases, TOPK expression was significantly correlated with multiple gastric cancer immune‐related molecules including PD‐L1 and IDO1. c) By Western blot analysis, TOPK knockdown was found to inhibit the overexpression of total STAT1, PD‐L1, and IDO1 proteins in gastric cancer cells under IFN‐γ stimulation, which specifically suppressed the overexpression of membrane‐bound PD‐L1 and cytosolic IDO1 in these cells. d) TOPK knockdown increased PBMC‐T cytotoxicity in gastric cancer cells after 48 h. e) TOPK knockdown inhibited l‐kynurenine expression under IFN‐γ and L‐Tryptophan (100um, L‐TRP) conditions. f) The Transwell experiment involving the co‐culture of gastric cancer cell lines and immune cells for 48 h was conducted. Each group had five experimental replicates. g) TOPK expression in gastric cancer cells (green) influences macrophage (orange) phagocytosis of tumor spheroids for 48 h. h) TOPK expression in gastric cancer cells (blue) influenced the killing activity of NK cells (purple) against tumor spheroids for 48 h. i) TOPK knockdown in 615‐derived MFC inhibits growth in vivo in 615 immunocompetent mice. Gastric cancer cells were treated with 10 ng mL^−1^ IFN‐γ for 48 h to simulate the IFN‐γ condition. Experiments were performed independently three times in vitro and at least six times in vivo, and a representative image is shown. Error bars represent the mean±SD of independent replicates. *p‐values* were determined using unpaired two‐tailed Student's *t*‐tests. ^*^ *p* < 0.05, ^**^ *p* < 0.01, ^***^ *p* < 0.001.

The proliferative and invasive effects of TOPK explain the phenomenon of GC inhibition by TOPK inhibitors OTS964 and OTS514 (Figure 1e). However, how OTS964 and OTS514 regulated IFN‐γ induced PD‐L1 adaptive expression and enhanced immune cells cytotoxicity was unclear (Figure 1g,i). Thus, we explored and analyzed TOPK's potential role in the immune metabolic microenvironment of GC. In a post‐immunotherapy cohort of patients with GC (ERP107734),^[^ ^28^ ^]^ the responsive group exhibited higher *TOPK* expression than the non‐responsive group. The area under the curve (AUC) for predicting immunotherapy response using the combination of PD‐L1 and TOPK expression was 0.876 (**Figure** **2**a). Additionally, *TOPK* expression in the group responding to immunotherapy was significantly elevated in cohorts from Pender et al.,^[^ ^29^ ^]^ IMvigor 210,^[^ ^30^ ^]^ and Rose et al.,^[^ ^31^ ^]^ with high AUCs (Figure S5a, Supporting Information). *TOPK* showed a significant correlation with immune infiltration correlation in both TCGA‐STAD and ACRG data (Figure S5b, Supporting Information). Further analysis of gene expression correlations revealed that TOPK expression was positively associated with multiple immune‐related molecules in gastric cancer. Notably, PD‐L1 and IDO1 (both established IFN‐γ–regulated immune checkpoints that influence immune evasion and therapeutic response) in gastric cancer cells showed significant positive correlations with TOPK (Figure 2b).

Next, because JAK2 (downstream kinase of IFN‐γ pathway) could phosphorylate and stabilize TOPK,^[^ ^11^ ^]^ we observed that upon IFN‐γ stimulation, phosphorylation of TOPK at the Tyr‐74 site and STAT1 at Tyr‐701 was increased, accompanied by increased protein levels of PD‐L1 and IDO1 (Figure S5c, Supporting Information), and PD‐L1 membrane translocation (Figure S5d, Supporting Information). Furthermore, we confirmed that *TOPK* depletion decreased STAT1, IDO1, and membrane‐bound PD‐L1 protein levels (Figure 2c). Initially, IFN‐γ (10 ng mL^−1^) did not exhibit cytotoxic effects on GC cells (Figure S5e, Supporting Information), with a 10: 1 effector‐to‐target ratio (E: T, PBMC‐T: BGC823) (Figure S5f, Supporting Information). Next, in the 2D Incucyte monitoring system, it was shown that PBMC‐T cells cytotoxicity was significantly enhanced by *TOPK* depletion in GC cells (Figure 2d) and attenuated by *TOPK* overexpression (Figure S5g, Supporting Information). In addition, IDO1 is responsible for consuming tryptophan (the nutritional substance of cytotoxic cells) and generating L‐Kynurenine (L‐KYN).^[^ ^32^ ^]^ In vitro, *TOPK* depletion resulted in decreased L‐Kynurenine production, as detected by immunofluorescence (IF) assays (Figure 2e). The knockdown of either PD‐L1 or IDO1 expression within BGC823 and NCI‐87 cells significantly reversed the inhibitory influence of TOPK overexpression on the secretion of GZMB and Perforin by PBMC‐T cells (Figure S5h, Supporting Information). We further performed Transwell assays under the condition of IFN‐ γ and L‐tryptophan (L‐TRP), which showed *TOPK* expression had influenced on the migration of immune cells, including PBMC‐T, NK‐92MI, and Phorbol 12‐Myristate 13‐Acetate (PMA) stimulated THP‐1 cells (Figure 2f). Further, in 3D model, macrophages (PMA stimulated THP‐1) (Figure 2g) and NK‐92MI (Figure 2h) exhibited significantly elevated cytotoxicity against GC cells with *TOPK* deficiency, and vice versa. Consistent with these findings, *TOPK* depletion in MFC significantly inhibited the xenograft growth of subcutaneous cells in a syngeneic mouse model (Figure 2i).

### 2.5 TOPK Enhances *STAT1* mRNA Translation Via Phosphorylating eIF4F Complex Components

**Figure 3:**
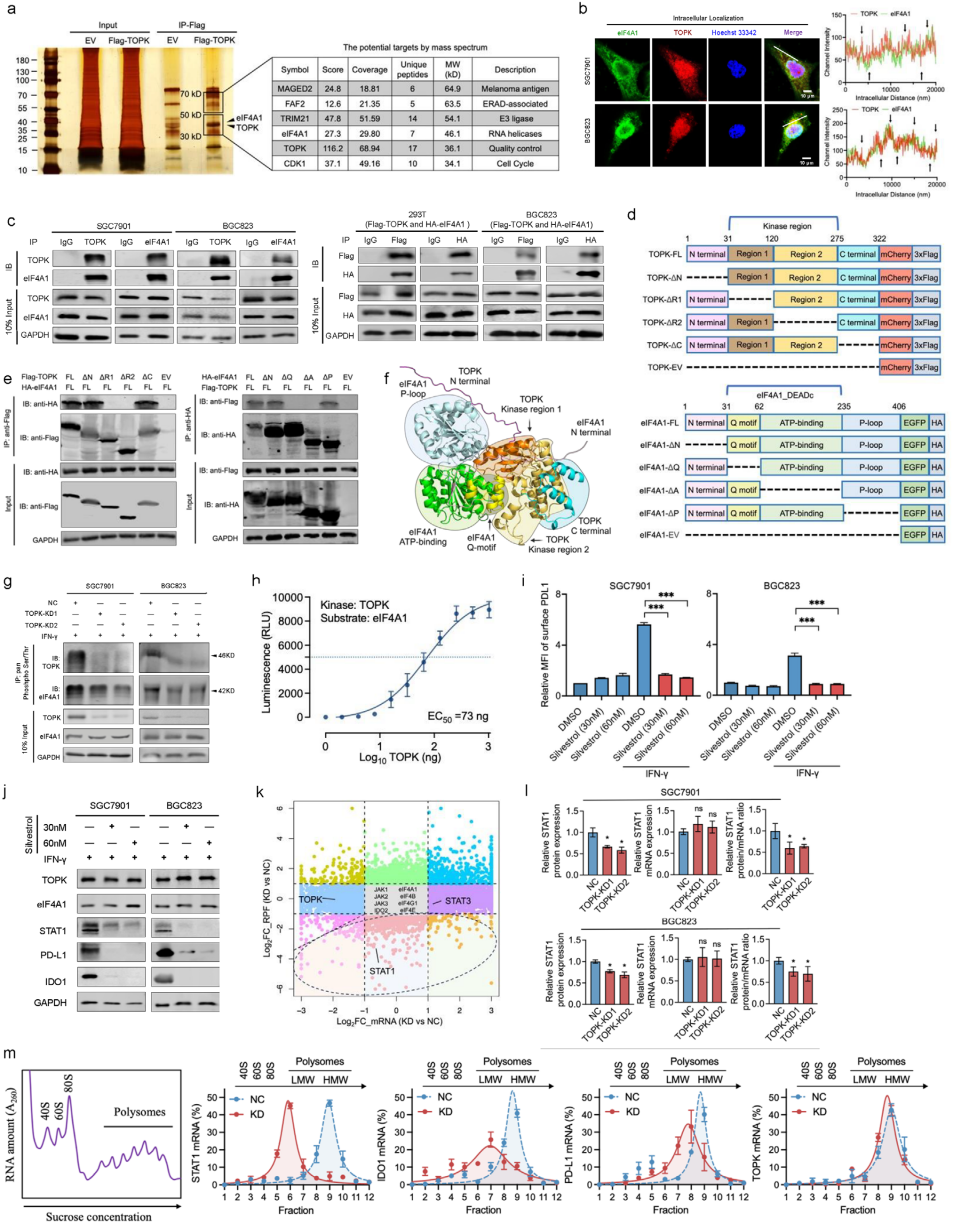
TOPK enhances *STAT1* mRNA translation via phosphorylating eIF4F complex components. a) Silver staining for mass spectrometry reveals potential TOPK‐interacting proteins, including eIF4A1 and CDK1. b) Intracellular co‐localization of TOPK and eIF4A1 in gastric cancer cells. c) The co‐immunoprecipitation (co‐IP) of endogenous TOPK and eIF4A1 proteins in SGC7901 and BGC823 cells, and co‐IP of exogenous TOPK and eIF4A1 in 293T and BGC823 cells. d) Annotation of different truncated mutations in TOPK and eIF4A1 proteins. e) Co‐IP assays involving TOPK and eIF4A1 with truncated mutations on one side. f) Molecular docking model of TOPK and eIF4A1. g) Co‐immunoprecipitation assay of TOPK and pan‐phosphorylated serine/threonine proteins. TOPK knockdown decreases eIF4A1 phosphorylation levels. h) In vitro kinase assay for TOPK‐mediated phosphorylation of eIF4A1. i,j) eIF4A1 functional inhibition by silvestrol (30, 60 nm) blocks IFN‐γ induced membrane PD‐L1 (i) and total STAT1, PD‐L1 and IDO1 (j) overexpression in gastric cancer cells. Functional inhibition of eIF4A1 did not affect TOPK expression. The dashed line represents the MFI of the DMSO with IFN‐γ group. k) Nine quadrant plots of differential RPFs via Ribo‐seq and mRNA expression via RNA‐seq in SGC7901 cells with TOPK knockdown (*n* = 3) compared to control cells (*n* = 4). l) TOPK knockdown downregulated *STAT1* translation efficiency (TE) in the protein/mRNA ratio, as confirmed by western blotting and qPCR in SGC7901 and BGC823 cell lines. m) Ribosome profiling assays reveal TOPK knockdown promotes *STAT1* mRNA decrease in ribosome polysomes under IFN‐γ conditions. *IDO1* and *PD‐L1* mRNA levels were only partially affected, whereas *TOPK* mRNA levels were not. Experiments were independently performed at least three times in vitro. Error bars represent the mean±SD of independent replicates. *p*‐values were determined using unpaired two‐tailed Student's *t*‐tests. ^*^ *p* < 0.05, ^**^ *p* < 0.01, ^***^ *p* < 0.001.

To understand how TOPK regulates IFN‐γ‐mediated remodeling of the immune microenvironment, we performed mass spectrometry‐based identification of TOPK interaction proteins, which included TRIM21, eIF4A1, and CDK1 (**Figure** **3**a). eIF4A1 is a crucial component of the eIF4F complex located in the eukaryotic cell ribosome and participates in RNA unwinding and translation through its DEAD domain.^[^ ^33^ ^]^ TOPK phosphorylation appeared to impact several pathways, including “translation activator activity” (Figure S3g, Supporting Information). Importantly, we identified the DEAD domain as a potential domain interacting with TOPK phosphorylation (Figure S3i, Supporting Information), and characterized the co‐localization of TOPK and eIF4A1 in GC cells (Figure 3b). The interaction between eIF4A1 and TOPK was validated both endogenously and exogenously (Figure 3c), and the kinase region of TOPK and the DEADc domain of eIF4A1 were mapped for their interaction (Figure 3d,e). By integrating these clues, molecular docking was performed, and eIF4A1 was suggested to bind to the active pocket of TOPK, providing structural conditions for phosphorylation (Figure 3f). Phosphorylation of eIF4A1 decreased upon *TOPK* depletion (Figure 3g). In vitro kinase assay confirmed that the active purified TOPK protein can directly phosphorylate eIF4A1 (Figure 3h).

We further analyzed the phosphorylation sites, scores, and rankings of 303 serine/threonine kinases of the eIF4F complex using the human serine/threonine kinome substrate atlas.^[^ ^34^ ^]^ Kinome analysis revealed that many phosphorylation sites on eIF4A1 were subject to strong phosphorylation by TOPK (including S78, T158, T393, and S56), ranking prominently among the kinases examined (Figure S6a, Supporting Information). Strikingly, many key eIF4F complex components were potential substrates with high phosphorylation scores and ranks, with predicted phosphorylation sites promoting translation, such as eIF4B‐S422 and eIF4E‐S209 (Figure S6a, Supporting Information). Therefore, TOPK binding to eIF4A1 may bring TOPK closer to other eIF4F complex components, creating conditions for further phosphorylation. We suggest that TOPK is involved in widespread phosphorylation of the eIF4F complex (Figure S6b, Supporting Information).^[^ ^17^ ^]^

We then explored whether eIF4A1 plays a central role in mediating TOPK functions in IFN‐γ stimulation. To this end, *eIF4A1* depletion (Figure S6c, Supporting Information) resulted in decreased IFN‐γ induced membrane PD‐L1 levels, accompanied by decreased expression of STAT1, PD‐L1, and IDO1 (Figure S6c–e, Supporting Information). Furthermore, the eIF4A1 inhibitor, silvestrol, decreased membrane PD‐L1 expression (Figure 3i) and total STAT1, PD‐L1, and IDO1 protein expression upon IFN‐γ stimulation (Figure 3j). Co‐localization of TOPK with eIF4E (Figure S6f, Supporting Information) or eIF4B (Figure S6g, Supporting Information) was also observed, with a mild effect on their intensity compared to that of eIF4A1 (Figure 3b). We performed Ribo‐seq assays and revealed decreased ribosome‐protected fragments (RPFs), including *STAT1*, *PD‐L1*, *IDO1* (Figure S6h, Supporting Information). Combined analysis of Ribo‐seq and transcriptome data revealed that *TOPK* depletion significantly decreased TE (ratio of RPF to mRNA), of *PD‐L1*, *STAT1*, and *IDO1* (Figure 3k). Proteomap analysis of down‐regulated transcription factors (TF) within the dRPF (Table S2, Supporting Information) exhibited “STAT1” in the purple module, indicating that STAT1 was an important TF whose expression decreased following *TOPK* downregulation in GC cells.

To elucidate the impact of TOPK on the RPFs of *STAT1* and *IDO1*, which exhibited a significant decrease in RPFs, we analyzed their sequences and open‐reading frames (ORFs). After *TOPK* knockdown, some ORFs of *STAT1*, including the coding DNA sequence (CDS) ORF, retained intron (RI) ORF, downstream ORF (dORF), novel ORF, and nonsense‐mediated mRNA decay (NMD) ORF, were not detected. In contrast, the CDS ORF of *IDO1* remained detectable. This indicates that the regulatory influence of TOPK on *IDO1* RPFs may not be as complete as that observed for *STAT1* (Figure S6j, Table S3, Supporting Information). *STAT1* mRNA and protein levels and TE was calculated (ratio of protein to mRNA), confirming that *STAT1* mRNA was not regulated, but protein and TE levels decreased upon *TOPK* knockdown (Figure 3l). Ribosome profiling assays showed that the RPFs of *STAT1*, *IDO1*, and *PD‐L1* decreased in the fractions of high‐molecular‐weight (HMW) polysomes (Figure 3m; Figure S6k, Supporting Information).

### 2.6 TOPK Inhibitor OTS964 Blocks IFN‐γ Induced PD‐L1 and IDO1 Expression

**Figure 4:**
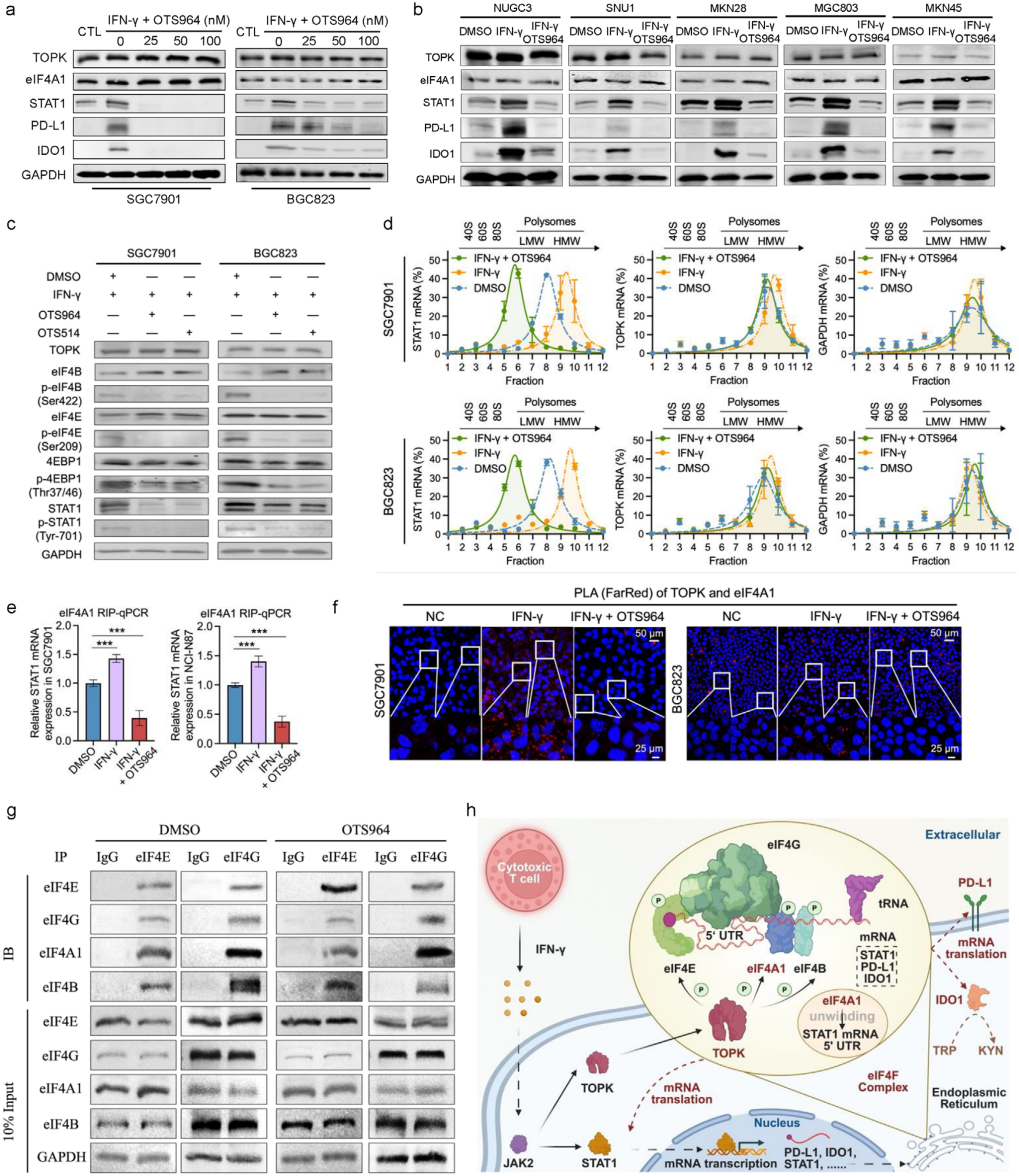
TOPK inhibitor OTS964 blocks IFN‐γ induced PD‐L1 and IDO1 expression. a) OTS964 decreased IFN‐γ induced total STAT1, PD‐L1 and IDO1 protein overexpression. TOPK and eIF4A1 protein expression levels were not affected. b) OTS964 decreases IFN‐γ induced total STAT1, PD‐L1, IDO1 protein overexpression in gastric cancer cells (NUGC3, SNU1, MKN28, MGC803, MKN45). TOPK and eIF4A1 protein expression levels were not affected. c) OTS964 and OTS514 (50 nm) decreased the phosphorylation levels of proteins, including p‐eIF4B‐Ser422, p‐eIF4E‐Ser209, p‐4EBP1‐Thr37/46, p‐STAT1‐Tyr701, and total proteins, including PD‐L1 and IDO1, in cancer cells. d) Ribosome profiling assays reveal TOPK inhibition via OTS964 promotes *STAT1* mRNA decrease in ribosome polysomes under IFN‐γ condition, but not for *TOPK* and *GAPDH* mRNAs in SGC7901 and BGC823. e) RNA binding protein immunoprecipitation‐qPCR assays (RIP‐qPCR) for analyzing the effect of TOPK inhibition on the binding of eIF4A1 protein to STAT1 mRNA. f) Proximity ligation assay (PLA) of spatial proximity interactions between TOPK and eIF4A1 in gastric cancer cells. Positive interaction (red) of TOPK and eIF4A1 was shown under IFN‐γ conditions in SGC7901 and BGC823. g) Co‐IP assay for analyzing the effect of TOPK inhibitor OTS964 on the formation integrity of the eIF4F complex. h) Schematic diagram of the mechanism by which cytoplasmic TOPK in tumor cells phosphorylates the eIF4F complex and increases the protein expression of STAT1, PD‐L1, and IDO1. Cancer cells were treated with 50 nm OTS964 for 48 h. Gastric cancer cells were treated with 10 ng mL^−1^ IFN‐γ for 48 h to simulate an IFN‐γ condition. Experiments were independently performed at least three times in vitro. Error bars represent the mean±SD of independent replicates. *p*‐values were determined using unpaired two‐tailed Student's *t*‐tests. ^*^ *p* < 0.05, ^**^ *p* < 0.01, ^***^ *p* < 0.001.

We observed that OTS964 administration attenuates proteins, including STAT1, PD‐L1, and IDO1, rather than TOPK and eIF4A1 under IFN‐γ stimulated conditions (**Figure** **4**a,b). Additionally, OTS964 inhibited IFN‐γ induced elevated membrane PD‐L1 (Figure 1i), accompanied with decreased p‐eIF4B‐S422, p‐eIF4E‐S209, p‐4EBP1‐T37/46 and p‐STAT1‐T701 (Figure 4c). OTS964 inhibited IFN‐γ induced enrichment of *STAT1*, but not *TOPK* and *GAPDH*, mRNA RPFs in HMW polysomes (Figure 4d). RNA‐protein immunoprecipitation‐qPCR (RIP‐qPCR) assay revealed that OTS964 inhibits the binding of eIF4A1 protein to STAT1 mRNA (Figure 4e). Subsequently, proximity ligation assay (PLA) experiments showed that OTS964 disrupted the binding of TOPK and eIF4A1 (Figure 4f). The co‐IP assay results showed that the components eIF4E, eIF4G, eIF4A1, and eIF4B still bind to each other at the protein level in the presence of OTS964, suggesting that TOPK inhibition does not alter the formation of the eIF4F complex (Figure 4g).

### 2.7 The Key Phosphorylation Sites of the eIF4F Complex Components Mediate the Translational Regulatory Effect of TOPK

To further identify the key phosphorylation sites in the eIF4F complex components that mediate the translational regulatory effect of TOPK, we constructed cell lines (SGC7901, NCI‐N87) harboring activating or inactive point mutations targeting the potential phosphorylation sites predicted in Figure S6a (Supporting Information) (eIF4A1‐S78, eIF4A1‐T158, eIF4A1‐S394, eIF4B‐S422, eIF4E‐S209). Based on these cell lines, we performed a series of experiments including Ribosome Profiling, RIP‐qPCR, and ELISA. The results confirmed that the phospho‐inactive mutations S78A and T158A of eIF4A1 reduced the ribosomal translation efficiency of *STAT1* mRNA in tumor cells, but had no effect on *PD‐L1* and *IDO1* (Figure S7a, Supporting Information). At the transcriptional mRNA level, the inactive mutations did not affect *STAT1* mRNA, but significantly decreased the transcriptional expression of P*D‐L1* and *IDO1* mRNA—downstream target genes of STAT1 protein (Figure S7b, Supporting Information). At the protein expression level, the inactive mutations led to a significant reduction in the levels of STAT1, PD‐L1, and IDO1 (Figure S7c, Supporting Information). For the phospho‐activating mutations S78E and T158E of eIF4A1, the results showed that the activating mutations increased the ribosomal translation efficiency of *STAT1* mRNA and reversed the decrease induced by TOPK inhibition; however, they had no significant effect on the ribosomal translation efficiency of *PD‐L1* and *IDO1* mRNA (Figure S7d, Supporting Information). At the mRNA transcriptional level, the activating mutations had no effect on *STAT1*, but increased its downstream targets *PD‐L1* and *IDO1*, and partially reversed the TOPK inhibition effect (Figure S7e, Supporting Information). At the protein expression level, the activating mutations ultimately caused a significant increase in STAT1, PD‐L1, and IDO1, and partially reversed the TOPK inhibition effect (Figure S7f, Supporting Information). However, neither the activating mutation S394E nor the inactive mutation S394A of eIF4A1 had a significant effect on the ribosomal translation efficiency or mRNA transcriptional expression of *STAT1*, *PD‐L1*, and *IDO1* (Figure S7g,h, Supporting Information). Finally, RIP‐qPCR experiments further directly confirmed that the phosphorylation status of S78 and T158 in eIF4A1 affects the binding of eIF4A1 protein to *STAT1* mRNA (Figure S7i–k, Supporting Information).

Next, we analyzed the role of other components of the eIF4F complex in the TOPK‐eIF4F pathway. The results showed that the phospho‐inactive mutations S422A of eIF4B and S209A of eIF4E caused a slight decrease in the ribosomal translation efficiency of PD‐L1 and IDO1 mRNA (with a reduction in fraction of no more than 1.5 units), with no change in STAT1 (Figure S8a, Supporting Information). In contrast, the phospho‐activating mutations S422E of eIF4B and S209E of eIF4E led to a slight increase in the translation efficiency of PD‐L1 and IDO1 mRNA (with an increase in fraction of no more than 1 unit), while STAT1 showed no significant change (Figure S8b, Supporting Information). Additionally, in the presence of Silvestrol, an eIF4A1 inhibitor, the activating mutations failed to reverse the Silvestrol‐induced decrease in the ribosomal translation efficiency of STAT1 mRNA (Figure S8c,d, Supporting Information). Finally, RIP‐qPCR experiments confirmed that neither the phospho‐inactive nor phospho‐activating mutations of eIF4B‐S422 and eIF4E‐S209 affect the direct binding between eIF4A1 protein and STAT1 mRNA (Figure S8e, Supporting Information). In summary, the diagram depicted the IFN‐γ‐TOPK‐eIF4F‐STAT1‐PD‐L1/IDO1 axis in GC (Figure 4h).

### 2.8 TOPK Inhibitor OTS964 Improves the Microenvironment and Enhances T Cell Function

**Figure 5:**
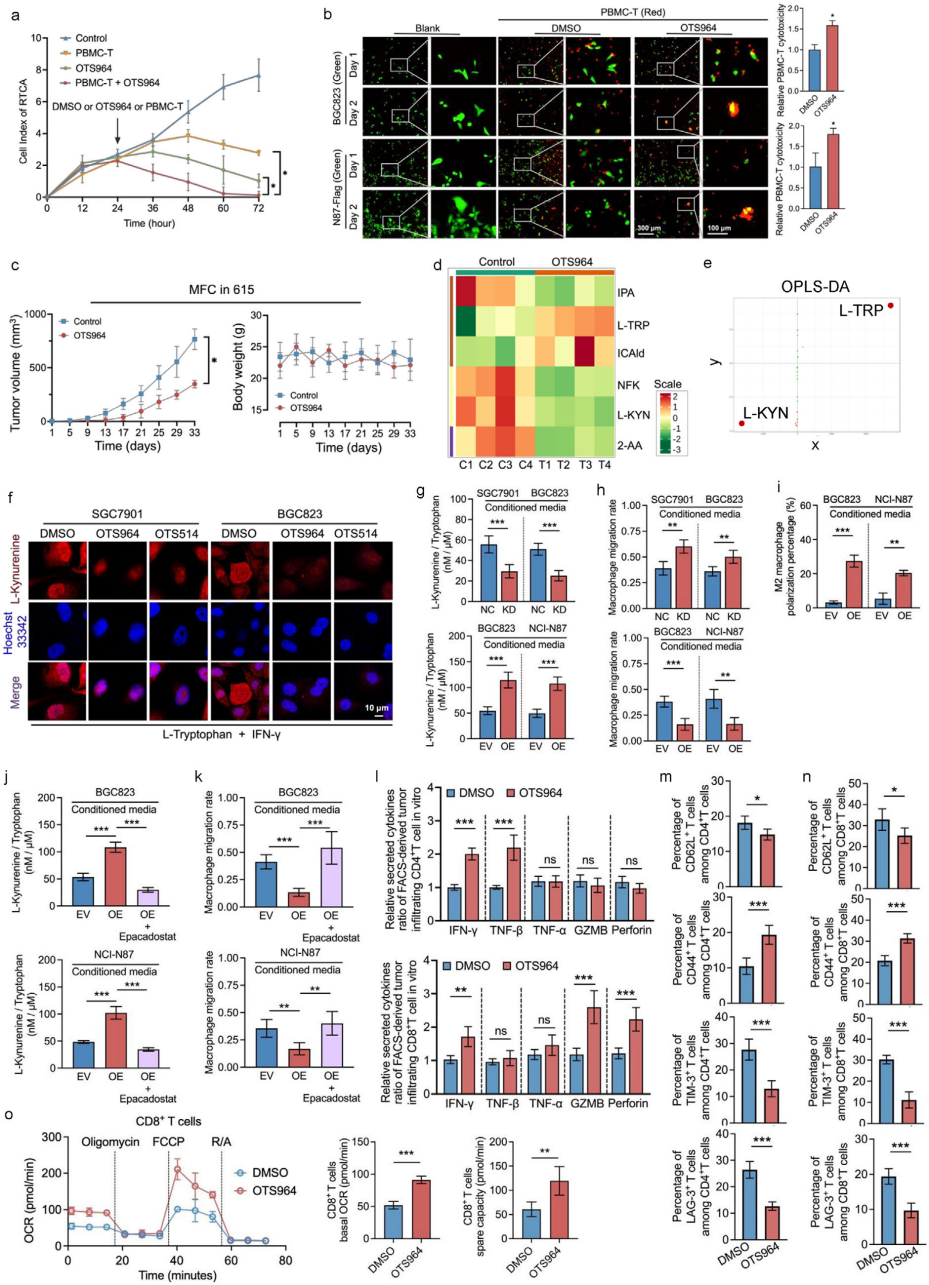
TOPK inhibitor OTS964 improves the microenvironment and enhances T cell function. a) The cell index of the non‐labeled real‐time cell analysis (RTCA) model, including gastric cancer cells, PBMC‐T, and compounds. OTS964 synergistically promoted PBMC cytotoxicity against gastric cancer cells (E: T = 5:1). b) OTS964 increased PBMC‐T cytotoxicity at 48 h against BGC823 and NCI‐N87 cells overexpressing TOPK in vitro. c) OTS964 inhibited subcutaneous cell allograft growth in vivo in 615 immunocompetent mice (*n* = 6 replicates). d) Metabolite assays of the tryptophan metabolic pathway in vivo treated with OTS964. OTS964 increased L‐tryptophan (L‐TRP) levels and reduced L‐Kynurenine (L‐KYN) levels in gastric cancer tumors (*n* = 4 replicates). e) In the OPLS‐DA S‐plot, the *x*‐axis represents the covariance between the principal components and metabolites, whereas the *y*‐axis represents the correlation coefficient between the principal components and metabolites. Metabolites closer to the upper‐right and lower‐left corners indicate more significant differences. Red points indicate metabolites with Variable Importance in Projection (VIP) values greater than 1, whereas green points represent metabolites with VIP values less than or equal to 1. f) OTS964 and OTS514 blocks IFN‐γ induced L‐Kynurenine metabolism. g) ELISA detection of kynurenine and tryptophan in the conditioned medium from tumor cells supernatants. h) TOPK expression in tumor cells suppresses the Transwell migration of macrophages. i) TOPK expression in tumor cells promotes M2 polarization of macrophages. j) The increased kynurenine/tryptophan ratio induced by TOPK expression in tumor cells can be reversed by the IDO1 inhibitor Epacadostat. k) The decreased macrophage migration rate induced by TOPK expression in tumor cells can be reversed by the IDO1 inhibitor Epacadostat. l) Detection of cytokines production of IFN‐γ, TNF‐β, TNF‐α, GZMB, and Perforin in CD4⁺ and CD8⁺ T cells sorted after tumor treated with TOPK inhibition or DMSO. m,n) Flow cytometry analysis of the membrane expression of CD44, CD62L, TIM3, and LAG3 on CD4⁺ T cells (m) and CD8⁺ T cells (n) derived from tumor cells. o Seahorse mitochondrial stress test verified that TOPK inhibition in tumor tissues enhances the basal metabolic function and reserve capacity of CD8^+^ T cells. Cancer cells were treated with 50 nm OTS964 for 48 h. Cancer cells were treated with 10 ng mL^−1^ IFN‐γ for 48 h to simulate IFN‐γ condition. Experiments were independently performed at least three times in vitro. Error bars represent the mean±SD of independent replicates. *p*‐values were determined using unpaired two‐tailed Student's *t*‐tests. ^*^ *p* < 0.05, ^**^ *p* < 0.01, ^***^ *p* < 0.001.

We employed a real‐time cell‐monitoring model to assess the regulatory effects of OTS964 on the cytotoxicity of PBMC‐T cells. OTS964 significantly enhanced the synergistic cytotoxic effects of PBMC‐T on SGC7901 cells, surpassing the efficacy of OTS964 or PBMC‐T alone (**Figure** **5**a). OTS964 also significantly enhanced PBMC‐T (red) cytotoxicity against BGC823 (green) and TOPK overexpressed NCI‐N87 cells (green) (Figure 5b). Consistent with these findings, OTS964 inhibited tumor growth of MFC‐CDA in immunocompetent mice (Figure 5c). Moreover, mass spectrum analysis of metabolites demonstrated that OTS964 increased L‐TRP and decreased L‐KYN production, blocking the L‐TRP metabolic pathway (Figure 5d,e). Confocal microscopy experiments confirmed that OTS964 inhibits TOPK, leading to reduced kynurenine production in tumor cells (Figure 5f). TOPK expression increases the ratio of kynurenine to tryptophan in the conditioned medium of tumor cells (Figure 5g), and suppresses the migration of macrophages in the Transwell model (Figure 5h), and promotes M2 polarization of macrophages (Figure 5i). However, the increased kynurenine to tryptophan ratio and the migration rate of macrophage induced by TOPK expression can be reversed by the IDO1 inhibitor Epacadostat (Figure 5j,k). In the CDX model treated with OTS964, inhibition of TOPK in tumor tissues increases IFN‐γ, TNF‐β secretion by sorted CD4⁺ T cells, and IFN‐γ, GZMB, Perforin secretion by sorted CD8⁺ T cells (Figure 5l). After TOPK inhibition, the proportions of LAG‐3‐positive and TIM‐3‐positive cells among CD4⁺T and CD8⁺T cells were decreased. The upregulated expression of CD44 on T cells indicated increased T cell activation, while the expression of CD62L showed a slight decrease (Figure 5m,n). For analyzing the metabolic state of CD8^+^ T cells isolated from tumors treated with OTS964, Seahorse mitochondrial stress tests were performed. TOPK inhibition in tumor tissues enhances the basal metabolic function and reserve capacity of CD8+ T cells (Figure 5o). These findings collectively suggest that the TOPK inhibitor OTS964 blocks IFN‐γ induced PD‐L1 and IDO1 expression in GC, thus alleviating the immunosuppressive effects of these immune checkpoints and enhancing the cytotoxicity and metabolic capacity of immune T cells in the immunometabolic microenvironment.

### 2.9 TOPK Inhibitor Synergizes with Immune Checkpoint Blockade for GC Therapy

**Figure 6:**
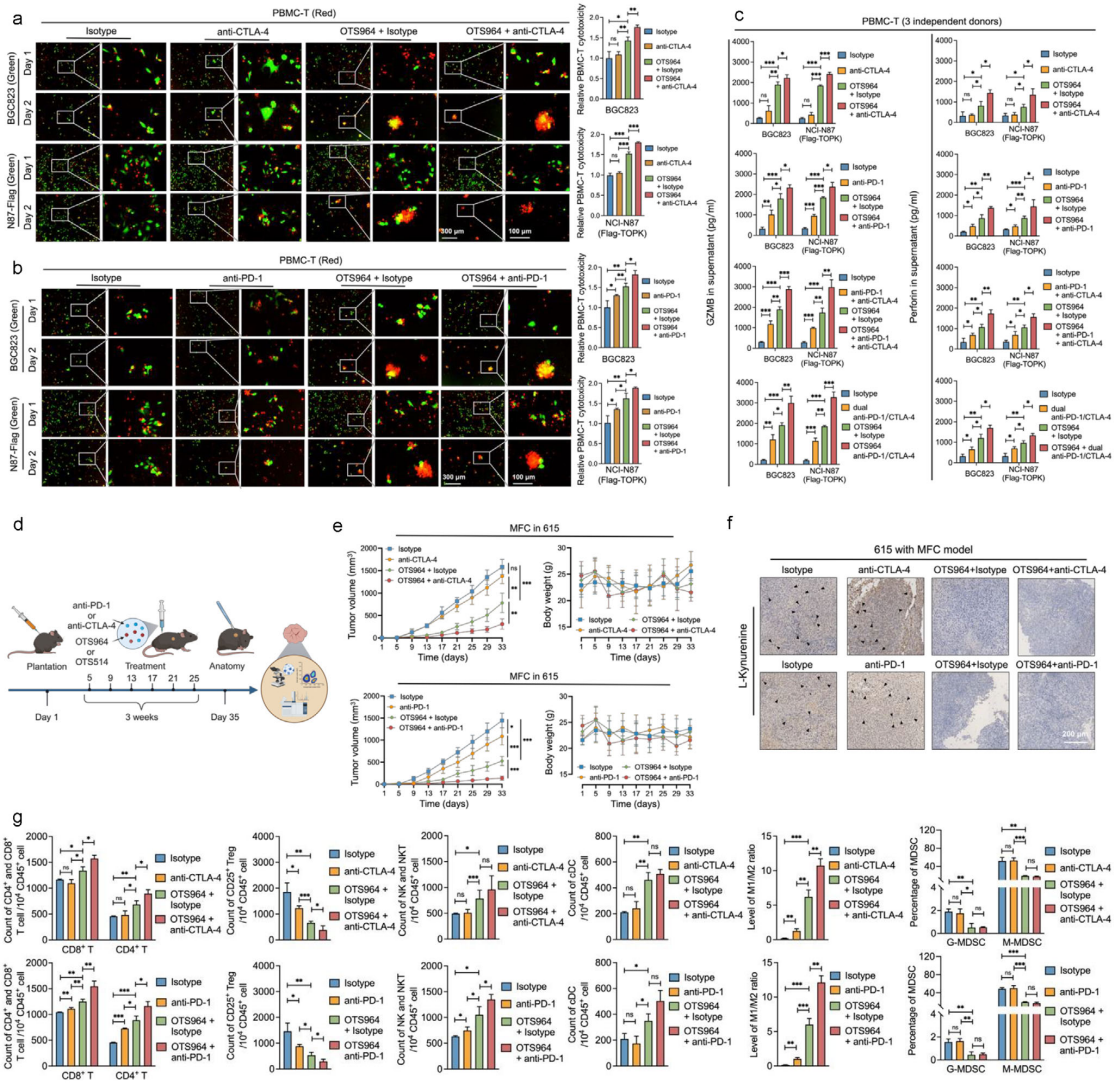
TOPK inhibitor synergizes with immune checkpoint blockade for gastric cancer therapy. a,b) Real‐time monitoring model for the co‐culture of labeled live cells reveals that the combination of the TOPK inhibitor OTS964 with anti‐CTLA‐4 (a) or anti‐PD‐1 (b) exhibits synergistic effects in increasing T cells cytotoxicity and inhibiting cell growth (BGC823 and NCI‐N87 with TOPK overexpression), anti‐CTLA‐4 antibody or isotype, 100 nm; and anti‐PD‐1 antibody or isotype, 20 nm. c) OTS964 alone or in combination with anti‐CTLA‐4 or anti‐PD‐1 antibodies significantly increased granzyme B or perforin secretion from PBMC‐T cells in a co‐culture model of gastric cancer cells. d) Model design of subcutaneous cell allografts in 615 immunocompetent patients treated with OTS964 alone or in combination with anti‐CTLA‐4 or anti‐PD‐1 antibodies. e) OTS964 or combined with anti‐CTLA‐4 or anti‐PD‐1 antibodies synergistically inhibited tumor growth in vivo in the 615 model. f) Immunohistochemical staining revealed that OTS964, in combination with anti‐CTLA‐4 or anti‐PD‐1 antibody, reduced the production of L‐Kynurenine. g) Multicolor flow cytometry assays revealed that OTS964 alone or in combination with anti‐CTLA‐4 or anti‐PD‐1 antibodies increased immune cell infiltration, including NK cells, NKT cells, CD4^+^ or CD8^+^ T cells, cDC cells, and the M1/M2 ratio in allografts, and CD25^+^ Tregs or myeloid‐derived suppressor cells (MDSC) were reduced. 50 nm OTS964 was used to treat cancer cells for 48 h. Gastric cancer cells were treated with 10 ng mL^−1^ IFN‐γ for 48 h to simulate IFN‐γ condition. Experiments were performed independently at least three times in vitro or five times in vivo, and one representative image is shown. Error bars represent the mean±SD of independent replicates. *p*‐values were determined using unpaired two‐tailed Student's *t*‐tests. ^*^ *p* < 0.05, ^**^ *p* < 0.01, ^***^ *p* < 0.001.

We evaluated the effects of TOPK‐targeted therapy with immune checkpoint inhibitors, including anti‐PD‐1, anti‐CTLA‐4, and dual anti‐PD‐1/CTLA‐4 antibodies, against GC. As expected, the anti‐immune therapies did not affect GC cells proliferation in an immunodeficient microenvironment (Figure S9a, Supporting Information). However, OTS964 enhanced PBMC‐T cytotoxicity against BGC823 or *TOPK* overexpressed NCI‐N87 cells, which was further amplified when combined with anti‐CTLA‐4 (**Figure** **6**a), anti‐PD‐1 (Figure 6b), or both (Figure S9b, Supporting Information). Our previous clinical trial using a dual anti‐PD‐1/CTLA‐4 bispecific antibody (cadonilimab) showed encouraging response rates,^[^ ^35^ ^]^ TOPK inhibitor combined with it exhibited promising synergistic effects (Figure S9c, Supporting Information). Meanwhile, OTS964 administration alone, or combined with immune checkpoint inhibitors, can markedly increase secretion of GZMB, perforin, and TNF‐β, but not TNF‐α (Figure 6c; Figure S9d, Supporting Information), indicating the potential role of OTS964 in promoting PBMC‐T cytotoxicity.

Next, we assessed the synergetic effect of OTS964 in combination with immunotherapy in vivo (Figure 6d) and observed that OTS964 inhibited subcutaneous xenografted GC tumor growth and further synergized with anti‐CTLA‐4 or anti‐PD‐1 antibodies (Figure 6e), accompanied by decreased L‐KYN (Figure 6f). Referring to the gating strategy of multicolor cytometry (Figure S9e, Supporting Information),^[^ ^36^ ^]^ our findings revealed that TOPK knockdown increased immune cell infiltration, including CD4^+^ or CD8^+^ T cells, NK cells, cDC cells, and the M1/M2 ratio, while CD25^+^ Tregs or myeloid‐derived suppressor cells (MDSC) were reduced (Figure S9f, Supporting Information). Similarly, OTS964 treatment increase the infiltration of cytotoxic immune cells, especially when administered in combination with anti‐PD‐1 or anti‐CTLA‐4 antibodies (Figure 6g).

### 2.10 TOPK is Associated with Clinical GC Malignant Phenotype

**Figure 7:**
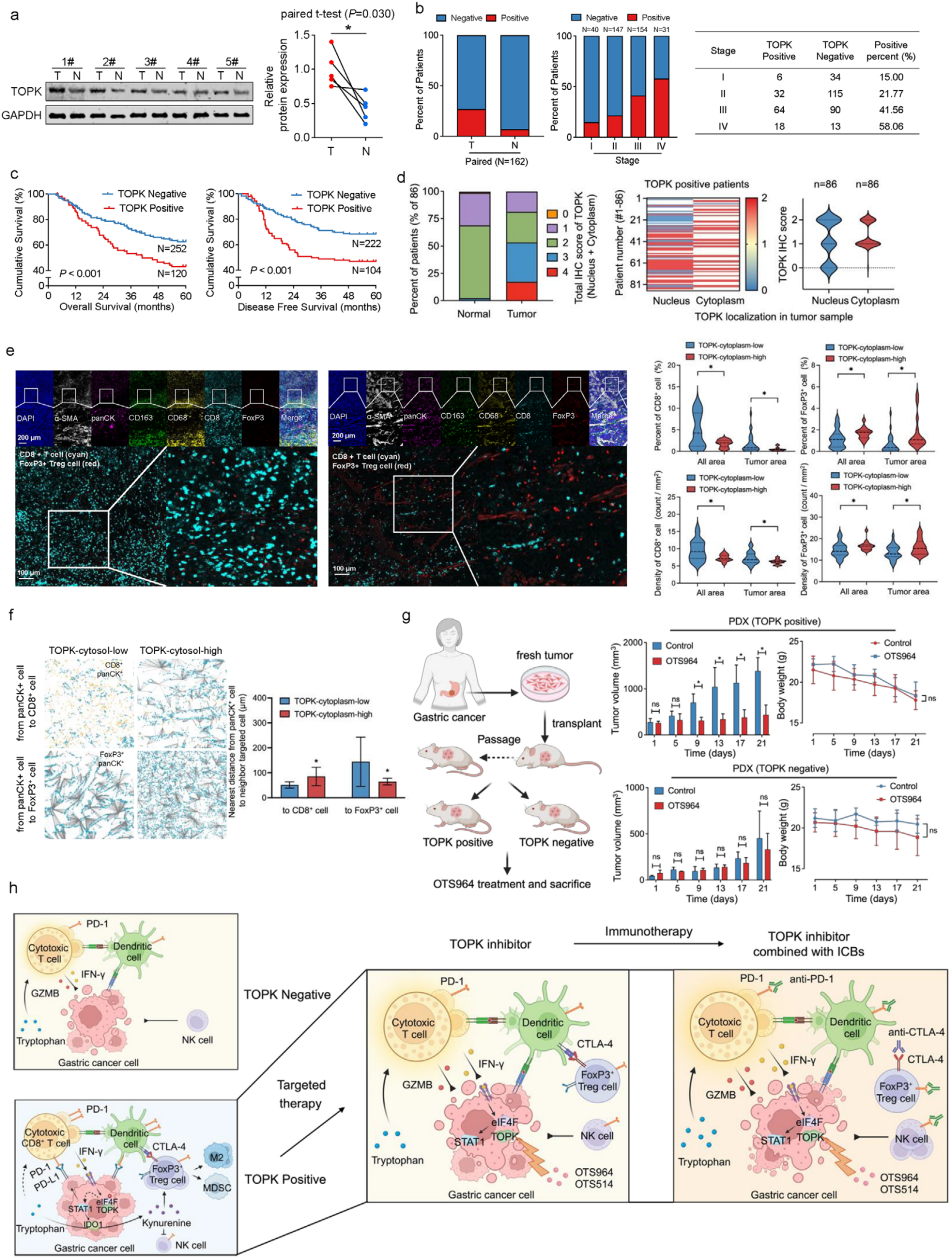
TOPK is associated with clinical gastric cancer malignant phenotype. a) Higher TOPK protein levels were detected via western blotting in tumor tissues compared to adjacent tissues of patients with gastric cancer (*n* = 5 patients). The paired two‐tailed Student's *t*‐test was performed. b) The positive rate of TOPK expression via immunohistochemical staining was higher in gastric cancer tumor tissues than in adjacent noncancerous tissues (*n* = 162 patients). The positivity rate for TOPK increased as the clinical stage progressed in patients with gastric cancer (*n* = 372 patients). c) TOPK‐positive patients with gastric cancer exhibited poor overall survival (*n* = 372 patients) or disease‐free survival (*n* = 326 patients). Kaplan–Meier survival curves and log‐rank tests were used. *p* < 0.05 is considered a significant difference. d) TOPK is commonly expressed in the tumor cell cytosol of gastric cancer tissues (*n* = 86). e) Multiplex immunohistochemistry (mIHC) assays of patients with gastric cancer demonstrated lower CD8^+^ T cell and FoxP3^+^ T cell infiltration in tumor tissues with high TOPK protein levels in the tumor cell cytosol (*n* = 45 patients). f) Spatial distance measurements of mIHC showed greater distances from panCK^+^ cells with high TOPK protein levels in the cytosol to neighboring CD8^+^ T cells and shorter distances to neighboring FoxP3^+^ T cells (*n* = 8 patients). g) A patient‐derived xenograft (PDX) model revealed that OTS964 inhibits the growth of subcutaneous TOPK‐positive tumor xenografts in vivo in NOD/SCID mice. h) Hypothetical diagram of the tumor microenvironment of gastric cancer in four scenarios including TOPK positive, TOPK negative, TOPK inhibitor, and TOPK inhibitor combined with immune checkpoint inhibitors (ICIs). Error bars represent the mean±SD of independent replicates of the mIHC assays. Experiments were performed independently at least three times in vitro and five times in vivo. *p*‐values were determined using unpaired two‐tailed Student's *t*‐tests. ^*^ *p* < 0.05, ^**^ *p*  < 0.01, ^***^ *p*  < 0.001.

We observed significantly higher TOPK levels in tumor tissues than in adjacent normal tissues (**Figure** **7**a). IHC staining (Figure S10a,b, Supporting Information) confirmed elevated TOPK expression in tumors (Figure 7b), correlating with advanced clinical stages. Overall, 32.26% patients with GC and 58.06% patients with TNM‐IV GC exhibited positive total TOPK protein expression (Figure 7b). Patients responding to immunotherapy for gastric cancer have a higher probability of high cytoplasmic TOPK expression in tumor cells before treatment (Figure S10c, Supporting Information). Survival analysis revealed that patients with high TOPK expression showed poorer overall survival (OS) and disease‐free survival (DFS) rates (Figure 7c). We validated findings using public databases (Pender et al.,^[^ ^29^ ^]^ Rose et al.,^[^ ^31^ ^]^ Braun et al.,^[^ ^37^ ^]^ and Liu et al.^[^ ^38^ ^]^), which indicated worse survival outcomes for patients with high TOPK expression (Figure S10d, Supporting Information).

We validated the regulatory role of cytoplasmic TOPK in clinical GC samples. The results showed the total score (nuclear and cytoplasmic) for TOPK expression in tumors was much higher than that in matched adjacent normal tissues (*n* = 86). Notably, most patients displayed widespread cytoplasmic TOPK expression, while some showed no nuclear expression (Figure 7d; Table S4, Supporting Information). Tumor tissues with high cytoplasmic TOPK expression exhibited decreased CD8^+^ cells and increased FoxP3^+^ T cells infiltration, as detected by mIHC assays (Figure 7e). Spatial distance analysis revealed tumor cells with high TOPK expression were more spatially distant from CD8^+^ cells but closer to FoxP3^+^ cells (Figure 7f). Patients with high cytoplasmic TOPK expression in tumor cells have a higher percentage and density of αSMA⁺ CAFs and CD163⁺ M2 macrophages in the tumor microenvironment (Figure S10e, Supporting Information). Moreover, PLA assays (Figure S10f, Supporting Information) showed spatial proximity between TOPK and eIF4A1 in tumor, not in adjacent normal tissues (Figure S10g, Supporting Information), accompanied by spatial proximity between TOPK and eIF4B or eIF4E in GC tumor (Figure S10h, Supporting Information). In pre‐clinical experiment, OTS964 significantly inhibited the percutaneous TOPK‐positive, but not TOPK‐negative, patient‐derived xenograft (PDX) model of GC (Figure 7g), suggesting TOPK inhibitors could alleviate GC growth autonomously. These results suggest cytoplasmic TOPK plays a pivotal role in remodeling immune microenvironment in GC (Figure 7 h; Figure S10i,j, Supporting Information).

## 3 Discussion

The current practical or potential approaches to targeting GC are limited, with HER2 accounting for 12–23%,^[^ ^39^ ^]^ CLDN18.2 for 17–18%,^[^ ^40^ ^]^ and FGFR1‐4 aberration accounting for 12% of cases.^[^ ^41^ ^]^ Through high throughput integrated CRISPR‐Cas9 and compound screening, we identified TOPK as another potential target. TOPK displayed high expression in 32.26% (120 out of 372 cases) of patients with GC and 58.06% of advanced stage IV patients. Therefore, targeted therapy against TOPK could cover a larger population of patients with GC, significantly inhibiting tumor malignancy. Importantly, TOPK has potential value in sensitizing immunotherapy by regulating the eIF4A1‐STAT1‐PD‐L1/IDO1 axis, resulting in immune cells exhaustion and tumor cells evasion, suggesting that TOPK is a potential druggable target for targeted therapy combined with immunotherapy for advanced GC.

The systematic research on druggable oncogenic kinases with dual roles in GC is lacking. It was reported TOPK inhibitor HI‐TOPK‐032 improves NK‐92MI cell infiltration into ovarian tumors, however the mechanism was not understood.^[^ ^42^ ^]^ Here, we identified the kinase TOPK as performing dual roles: regulating the cell cycle in the nucleus and performing uncharacterized functions in the cytoplasm. TOPK's cytoplasmic function involves mediating IFN‐γ‐induced PD‐L1 and IDO1 expression, thereby reshaping the immune microenvironment in GC.

It was validated that PD‐L1 overexpression triggered by IFN‐γ/JAK2/STAT1 pathway leads to tumor evasion from T cells through PD‐L1/PD‐1 interaction.^[^ ^43^, ^44^ ^]^ TOPK is also phosphorylated and activated by JAK2 in Burkitt Lymphoma cells.^[^ ^11^ ^]^ Therefore, we investigated the association between TOPK‐PD‐L1 and IFN‐γ/JAK2 in our study. We found TOPK‐mediated interaction and phosphorylation of eIF4A1 and other eIF4F complex members will influence STAT1 TE and downstream IFN‐γ induced PD‐L1 and IDO1 expression in GC (Figure S10i, Supporting Information). In summary, TOPK acts as a “power amplifier” for the JAK‐STAT pathway: TOPK plays a critical role in amplifying this pathway: activated by JAK2, TOPK further phosphorylates and activates the eIF4F complex. Among the key components of this complex, eIF4A1—after being phosphorylated and activated at S78 and T158 sites—specifically enhances the unwinding activity of the 5′UTR of STAT1 mRNA. This enhancement increases the translation efficiency of STAT1, leading to elevated STAT1 protein expression, which in turn significantly upregulates the transcriptional and protein expression levels of PD‐L1 and IDO1, the downstream target genes of STAT1. Nevertheless, eIF4B and eIF4E have no significant effect on STAT1, but exhibit a weak translational enhancing effect on PD‐L1 and IDO1. This effect has limited capacity to reverse the final reduction in PD‐L1 and IDO1 protein expression when eIF4A1 is inhibited.

Here, we showed that TOPK has potential therapeutic value in the immune metabolic microenvironment of GC. As shown in the hypothetical diagram (Figure 7h), tryptophan emerged as a crucial substance for the normal functioning of immune cells, facilitating their cytotoxic effects against GC cells. In TOPK‐expressing tumor tissues, the intertumoral cytoplasmic TOPK interacts with and phosphorylates the eIF4F component, eIF4A1, promoting the translation of *STAT1* mRNA, leading to high expression of PD‐L1 and IDO1 induced by immune cells secreted IFN‐γ. The immune checkpoints PD‐L1 and IDO1 promote immune evasion from cytotoxic cells. Elevated IDO1 in tumor cells metabolizes tryptophan into L‐kynurenine, reducing the availability of immune cells and subsequent exhaustion.^[^ ^45^ ^]^ Combining TOPK inhibitors with immune checkpoint inhibitors can have a synergistic effect in killing tumor cells (Figure 7h, right panel).

Several TOPK inhibitors, such as OTS964, have shown results in inducing complete tumor regression by inhibiting cytokinesis.^[^ ^4^ ^]^ Other inhibitors, including OTS514,^[^ ^46^ ^]^ have also demonstrated potent anti‐tumor effects. IFN‐γ‐JAK2‐STAT1 pathway inhibition is the optimal strategy to inhibit both PD‐L1 and IDO1 expression.^[^ ^24^ ^]^ TOPK inhibitors can intervene in this process by blocking the co‐expression of PD‐L1 and IDO1 and inhibiting the malignant potential of tumor cells. In summary, TOPK inhibitors can enhance the cytotoxic effect of immune cells by mediating the adaptive expression of IFN‐γ induced PD‐L1 and IDO1 by phosphorylating components of the eIF4F complex, including eIF4A1 at S78 and T158, eIF4B at S422, and eIF4E at S209, ultimately inhibiting immune cells cytotoxicity and promoting immune evasion of GC cells. We also provide evidence that TOPK inhibitors, in combination with immune checkpoint blockade for sensitizing GC cells, offer new therapeutic strategies for targeted therapy plus immunotherapy for TOPK‐positive GC.

## 4 Experimental Section

### Patients and Samples

We collected 372 GC patients’ specimens with clinicopathological records and survival outcomes from Peking University Cancer Hospital, diagnosed histopathologically, and untreated before surgery between 2009 and 2015. The other specimens were collected from 86 patients diagnosed with stomach adenocarcinoma, and matched tumor and normal adjacent tissue sample slides were provided, which underwent IHC and mIHC. All participants provided written informed consent in accordance with the Declaration of Helsinki. The Ethics Committee of Peking University, Beijing Cancer Hospital approved this study (2019KT111).

### Antibodies, Chemicals, Kits, Oligonucleotides, and Plasmids

The antibodies, chemicals, kits, oligonucleotides, and plasmids used here are listed in (Tables S5–S9, Supporting Information).

### CRISPR‐Cas9 Whole‐Genome Screening

CRISPR‐Cas9 screening combined with compound screening was performed to explore potential mediators in the IFN‐γ/PD‐L1 pathway in GC (NCI‐N87) under IFN‐γ conditions. This procedure was performed as previously described.^[^ ^21^ ^]^ The MAGeCK software package was used to quantify and statistically assess sgRNA and gene enrichment, which provided logarithmic fold changes for sgRNAs and genes, indicating the degree of enrichment of these elements within each cell population.^[^ ^47^ ^]^

### The Source of Public Data

Public data were used for the immunotherapy response analysis, including ERP107734,^[^ ^28^ ^]^ Pender et al.,^[^ ^29^ ^]^ IMvigor 210,^[^ ^30^ ^]^ and Rose et al.^[^ ^31^ ^]^ For external validation of the survival analysis, public data were used, including Pender et al.,^[^ ^29^ ^]^ Rose et al.,^[^ ^31^ ^]^ Braun et al.,^[^ ^37^ ^]^ and Liu et al.^[^ ^38^ ^]^

## Conflict of Interest

The authors declare no conflict of interest.

## Author Contributions

J.F.J., X.F.X., J.B.C., and L.T.H. conceived the study; J.B.C. and L.T.H. contributed to biospecimen processing; J.B.C., L.T.H., G.J.W., Q.Y., and Q.D.L. analyzed the data and images; J.B.C., G.J.W., X.Y.W., C.C., H.B.Z., X.Y.L., and Q.D.L. performed the experiments; J.B.C., G.J.W., L.T.H., Z.N.L., and X.H.T. performed bioinformatic analysis; X.F.X., L.T.H., Y.H., and T.G. contributed to collection of clinical samples; X.F.X. and L.T.H. contributed to critical review of data; J.B.C., X.F.X., and L.T.H. drafted and revised the manuscript; J.B.C., L.T.H., G.J.W., B.F., X.F.X., and J.F.J. supervised the study; and L.T.H., T.G., B.F., X.F.X., and J.F.J. obtained funding.

## Supporting information



Supporting Information

Supporting Tables

## Data Availability

The data that support the findings of this study are available on request from the corresponding author. The data are not publicly available due to privacy or ethical restrictions.
