# Supplementary material for: TOPK Inhibition Promotes Anti‐Tumor Immunity Via eIF4F Complex Mediated STAT1 Translation in Gastric Cancer
Source: Adv Sci (Weinh). 2025 Dec 19;13(13):e17380. doi: 10.1002/advs.202517380 (PMC12955895; doi:10.1002/advs.202517380)
Supplement: Supplementary file 1 — Supporting Information [file ADVS-13-e17380-s001.docx]

**SUPPLEMENTAL MATERIALS AND METHODS**

**Animal model and treatments of** **cell-derived xenograft (CDX), cell-derived allograft (CDA), and patient-derived xenograft (PDX)**

Male mice, including immunodeficient BALB/c-nude and immunocompetent 615 aged 4-6 weeks, were used to establish the CDX model. Mice were obtained from the Institute of Hematology and Blood Diseases Hospital of Peking Union Medical College. The mice were maintained under stringent conditions within a controlled, specific pathogen-free facility, and their care adhered strictly to animal welfare protocols. A single-cell suspension in PBS containing 1×10^5/100 ul cancer cells or PBS alone was injected subcutaneously into each mouse's flank region. Tumor growth was regularly monitored, weighed, and measured with volume calculated using the formula: V= (length×width×width)/2. Mice showing signs of distress were euthanized. Tumors were excised, measured, and further analyzed. Lung metastasis was established in a CDX mouse model using luciferase-transfected cells and an in vivo imaging system. Mice received an inhalational anesthetic, and the harvested cells were injected into the tail vein. Mice were monitored regularly for signs of distress, weight loss, and overall health, and tumor metastasis was assessed using bioluminescence imaging. Upon reaching the experimental endpoint or exhibiting signs of severe morbidity, the mice were euthanized, and the lungs were harvested for histological analysis. The metastatic burden in the lungs was quantified by measuring the bioluminescence signal intensity, and the metastatic nodules were counted in the excised samples. The PDX model was established as previously described ^1,2^. Immunodeficient NOD/SCID mice were used for this purpose.

Treatments for 6–8 weeks old animals included targeted therapies or combined immunotherapy. Mice were randomly divided into treated and control groups. OTS964 (Cat# S7648, Selleck, Houston, Texas, United States) diluted in DMSO was administered by intraperitoneal injection at a dose of 1 mg/injection, with an equal dilution ratio and volume of DMSO used for the control group. InVivoMAb anti-mouse PD-1 (Cat# BE0146, BioXCell, Lebanon, New Hampshire, USA) or isotype control (InVivoMAb rat IgG2a isotype control, anti-trinitrophenol, Cat# BE0089, BioXCell) diluted with InVivoPure pH 7.0 Dilution Buffer (Cat# IP0070, BioXCell) was administered by intraperitoneal injection at a dosage of 250ug/injection. InVivoMAb anti-mouse CTLA-4 (Cat# BE0131, BioXCell) or isotype control (InVivoMAb polyclonal Syrian hamster IgG, Cat# BE0087, BioXCell) diluted with buffer was also administered at a dosage of 250ug/injection. For combined therapies, the two diluents were mixed and administered via intraperitoneal injection. In the PDX model, the drug was administered three times per week. The dosing regimen for animal administration of the CDA model is shown (Fig. 5d). Mice were euthanized upon the occurrence of dermal necrosis or when the tumor volume approached 2,000 mm^3^. All animal experiments were conducted in strict accordance with ethical policies and procedures approved by the Animal Care and Use Committee of Peking University (permission number: EAEC 2019-03). All efforts were made to minimize pain and distress.

**Patient and Public Involvement**

In this study, no relevant work involving the participation of patients and the public was carried out.

**Cell lines**

The commercial cell lines (KATO-3/CVCL_0371, SNU1/CVCL_0099, NUGC3/CVCL_1612, HGC27/CVCL_1279, MFC/CVCL_5J48, AGS/CVCL_0139, MKN45/CVCL_0434, NCI-N87/CVCL_1603, SNU16/CVCL_0076, and CT26/CVCL_7254) were used. The exclusively owned cell lines (SGC7901, MKN28, MGC803, BGC823) owned by the Key Laboratory of Carcinogenesis and Translational Research (Ministry of Education) and Biological Sample Bank of Peking University Cancer Hospital (Beijing, China). KATO3, SNU1, HGC27, SNU16, MFC, AGS, NCI-N87, and CT26 cell lines were obtained from the National Collection of Authenticated Cell Cultures (Shanghai, China). NUGC3, MKN28, and MKN45 cells were obtained from the Japanese Collection of Research Biosources Cell Bank (Ibaraki City, Japan). The NK-92MI (CVCL_3755) cells were obtained from iCell (Shanghai, China). THP-1 (CVCL_0006) cells were obtained from Abm (Richmond, Canada). These cell lines underwent recent STR profiling with authentication reports and were tested to confirm the absence of contamination.

**Cell culture, transfection, and gene editing**

Cancer cell lines were cultured in Dulbecco's Modified Eagle Medium (DMEM) medium supplemented with 10% fetal bovine serum (FBS) and 1% penicillin-streptomycin under 37°C and 5% CO_2_ conditions. Adherent cells in the exponential growth phase were harvested using a trypsin-EDTA solution and resuspended in phosphate-buffered saline (PBS). Additionally, NK-92MI cells were cultured in a specific medium (Catalog #iCell-h331-001b, including MEMα medium, FBS, horse serum, inositol, β-mercaptoethanol, folic acid, penicillin-streptomycin, iCell, Shanghai, China). THP-1 cells were cultured in a Roswell Park Memorial Institute (RPMI)-1640 medium. Phorbol 12-myristate 13-acetate (PMA), 100 ng/ml, was used to stimulate the differentiation of THP-1 cells into macrophages for further studies. The siRNA or plasmid (shRNA or OE) targeting TOPK, eIF4A1, or the negative control was diluted in Opti-MEM. Cell transfection and gene editing followed the manufacturer's instructions. Lipofectamine 2000 (Thermo Fisher Scientific, Waltham, MA, USA) was used to transfect siRNA or plasmids into cells. Gene knockdown or overexpression efficiency was assessed using qPCR, and Western blotting (WB) was used to measure mRNA and protein levels.

**Isolation and culture of PBMC-T cells**

Peripheral blood samples were collected from human donors in ethylenediaminetetraacetic acid (EDTA) tubes by standard venipuncture. PBMC were isolated, and PBMC-T cells were enriched and cultured according to the manufacturers' instructions (RosetteSep™ Human T Cell Enrichment Cocktail, and ImmunoCult™ Human CD3/CD28 T Cell Activator, StemCell Technologies Inc., Vancouver, Canada; Ficoll-Paque™ Plus, Cytiva, Massachusetts, USA). PBMC-T cells were resuspended and assessed using a trypan blue exclusion assay. The cells were cultured for at least three days in RPMI-1640 medium supplemented with 10% FBS, 1% penicillin-streptomycin, 10 ng/ml IL-2, and 25 ul/ml CD3/CD28 activator.

**High content imaging (HCI) of 3D tumor spheroids**

A 3D tumor spheroid model was constructed via HCI to stimulate and evaluate cancer cell proliferation at the 3D level, according to the manufacturer's protocol for spheroid formation via Corning spheroid microplates (Cat. No. 4520; Corning, New York, USA) into which the tumor cells were seeded. Real-time monitoring was performed using a Cellvoyager CV8000 system (Yokogawa, Tokyo, Japan) and analyzed using high-content analysis software (CellPathfinder, Yokogawa, Tokyo, Japan). Briefly, in the brightfield assays for monoculture, 100 µL of cell suspension (5×10^4 cells / mL) in DMEM and 10% FBS were added. The size of the tumor spheroids was measured at 24, 48, and 72 hours. For co-culture assays in the dark-field, 50 µL of cancer cells were initially seeded into microplates at a density of 1×10^5 cells/mL at 0 h, and THP-1-derived macrophages or NK-92MI cells were added for co-culture at 6-12 hours. 3D cell viability was assessed using the CellTiter-Glo 3D Cell Viability Assay Kit (Promega, Madison, WI, USA), and luminescence was measured using a microplate reader (BioTek Synergy H1, Agilent, CA, USA) after 72h of monoculture or co-culture.

**Real-time monitoring model of live cells**

A real-time imaging monitoring model of live cells was used to evaluate cell proliferation, migration, and cytotoxicity using the Incucyte ZOOM system (Sartorius, Göttingen, Germany). In the bright field, incucytes were utilized to monitor unlabeled 2D monoculture models, and in the dark field, incucytes were used to monitor labeled 2D co-culture cell models. The cancer cells were green-labeled (Cytolight Rapid Green Dye, Sartorius, Göttingen, Germany) and masked in green, and T cells from peripheral blood mononuclear cells (PBMC-T) cells were red-labeled (Cytolight Rapid Red Dye, Sartorius, Göttingen, Germany) and masked in red or purple (Fig. 1f, S1e). A total of 3,000 tumor cells were added per well, and the PBMC-T: target ratio was 10:1. The real-time results of cell confluence were analyzed using the Incucyte ZOOM Software (Sartorius, Göttingen, Germany).

The xCELLigence Real-time Cell Analysis (RTCA, Agilent, Basel, Switzerland) utilizes specialized microelectronic sensor arrays integrated into the bottom of cell culture plates to detect cellular events such as attachment, proliferation, and cytotoxicity. Compared with other monitoring methods based on imaging, RTCA provides dynamic, time-resolved data, allowing for a more precise characterization of cellular responses to various stimuli or treatments. First, tumor cells in 50 μl DMEM medium were added to each well, and 50ul DMEM medium with OTS964 compounds or PBMC-T cells (E: T=5:1) or a combination were added at 24 hours. The cell index was recorded for 72 h, and continuous real-time results were analyzed using the xCELLigence RTCA Software 2.0 (Agilent, Basel, Switzerland).

**Confocal assay, immunofluorescence staining (IF), EdU/TUNEL assays, and JC1 assays**

Confocal and IF assays were performed as previously described ^3^, including subcellular protein or molecule visualization, EdU/TUNEL assays, and JC1 assays. Briefly, for immunofluorescence staining, cells were fixed and permeabilized with 0.1% Triton X-100 for 20 min. Non-specific binding was blocked at room temperature. Subsequently, the cells were incubated with primary antibodies against the target proteins. After washing with PBS, the cells were incubated with appropriate secondary antibodies conjugated to fluorophores (Alexa Fluor 488 for green fluorescence and Alexa Fluor 594 for red fluorescence) for 1 h at room temperature. The nuclei were counterstained with Hoechst-33342 for 5 min. Finally, the coverslips were mounted onto glass slides using a mounting medium and sealed. EdU/TUNEL or JC1 assays were conducted according to the manufacturer's instructions (Cell-LightTM EdUTP TUNEL Cell Detection Kit, RiboBio, Guangzhou, Guangdong, China; Enhanced mitochondrial membrane potential assay kit with JC-1; Beyotime, Nanjing, Jiangsu, China).

**Colony forming unit (CFU) assay**

CFU assays were performed to assess the ability of a single tumor cell to form colonies, as previously described ^3^. Briefly, 5×10^2 cells were seeded into each well of a 6-well culture plate in triplicate and incubated for 10-14 days to allow colony formation, adding interventions during this period or the preliminary phase.

**Transwell assay of migration and invasion**

CFU assays were performed to assess the ability of cancer cells to migrate and invade, following a previously described protocol ^4^. Briefly, a cell suspension of 5×10^4 cells in 200 μL of serum-free DMEM was seeded onto polycarbonate filters. For the invasion assay, the filters were pre-coated with 100 μL of Matrigel, while for the migration assay, they remained uncoated. The lower chamber was supplemented with 400 μL of DMEM containing 10% FBS. Continuous real-time results were analyzed using the Incucyte ZOOM Software (Sartorius, Göttingen, Germany).

**Wound healing assay**

Wound healing assays were performed to assess cell motility following a previously described protocol ^4^. Briefly, Wounds were induced by scratching monolayers of cells at full confluence utilizing Incucyte® 96-Well Woundmaker Tool (Sartorius, Göttingen, Germany). Subsequently, the growth medium was replaced with a serum-free medium. The wounds were monitored in real-time utilizing an Incucyte System and analyzed by Incucyte® Scratch Wound Analysis Software Module (Sartorius, Göttingen, Germany).

**Proximity ligation assay (PLA) assay**

PLA assays were performed to analyze the spatial proximity and direct interaction of molecules within cells, according to the manufacturers' instructions (Duolink® In Situ Detection Reagents FarRed, Sigma-Aldrich, St. Louis, Missouri, USA). In addition, TOPK protein was conjugated with fluorescein (CoraLite® Plus 488-conjugated PBK Polyclonal antibody, Proteintech, Chicago, Illinois, USA) for evaluating the rate of Green-FarRed proximity (Fig. S9d).

**Immunohistochemical staining (IHC)**

Excised tumors were fixed in 10% formalin, embedded in paraffin (Formalin-Fixed Paraffin-Embedded, FFPE), and sectioned for histological analysis using hematoxylin and eosin (HE) staining, following a previously described protocol ^5^, and the manufacturer's instructions (Dako REAL EnVision Detection System, Peroxidase/DAB+, Rabbit/Mouse; Dako, Carpinteria, USA).

**The multiplex IHC (mIHC)**

mIHC assays were performed to detect multiple proteins in the FFPE sections according to the manufacturer's instructions (AlphaTSA Multiplex IHC Kit, Alphaxbio, Beijing, China). Briefly, sections were incubated with primary antibodies against specific targets overnight at 4°C. After washing, the sections were incubated with horseradish peroxidase (HRP)-conjugated secondary antibodies for 1 h at room temperature. Signal amplification was performed using tyramide signal amplification (TSA) technology with appropriate fluorophore-conjugated tyramide reagents. The primary antibodies used are listed in the Supplemental Material. The nuclei were counterstained with DAPI (4',6-diamidino-2-phenylindole). Imaging was performed using a fluorescence microscope (ZEISS AXIOSCAN 7; Carl Zeiss, Oberkochen, Germany). Data analysis was performed using image analysis software (HALO software, Indica Labs, Corrales, New Mexico, USA) to quantify the fluorescence signals and co-localization of different markers within the tissue sections. In the spatial distance analysis, regions of interest (ROIs) were defined based on histological features, and the spatial distances between different cell populations were measured using built-in algorithms.

**Quantitative real-time reverse transcription PCR (qRT-PCR)**

Total RNA was extracted from cells using TRIzol Reagent (Thermo Fisher Scientific, Waltham, Massachusetts, USA), and RNA concentration and purity were assessed using a NanoDrop spectrophotometer (Thermo Fisher Scientific, Waltham, Massachusetts, USA). Reverse transcription of RNA into cDNA was performed using ABScript III RT Master Mix for qPCR with gDNA Remover (ABclonal, Hubei, Wuhan, China). qPCR reactions were performed using the Genious 2X SYBR Green Fast qPCR Mix (ABclonal, Hubei, Wuhan, China). Gene-specific primers are listed in supplemental materials (Tab. S8). The procedure was performed as per the manufacturer's instructions. qPCR amplification was performed using the 7500 Real-Time PCR System (Applied Biosystems, Foster City, California, USA). Gene expression levels were normalized to GAPDH and calculated using the 2^(-ΔΔCt). Each experiment was performed in triplicate at least, and data are presented as mean±SD.

**RIP-qPCR**

RNA immunoprecipitation (RIP) was conducted with RIP kit (abs50071, Absin Bioscience, Shanghai, China). 2×107 cells were collected, washed with PBS, 1/4 reserved as Input, and the rest lysed in Complete RIP Lysis Buffer with Protease/RNase Inhibitor followed by sonication and centrifugation to get supernatant lysis. Protein A/G Magnetic Beads were washed, incubated with 5 μg eIF4A1 antibody (IP group) or normal IgG (control) at room temperature for 2 h, then mixed with 900 μL RIP Buffer and 150 μL lysis, and incubated overnight at 4°C. After 5 washes, RNA in Input, IgG and IP fractions was purified via Trizol-chloroform extraction, isopropanol precipitation and 75% ethanol washing, then dissolved in DEPC. Purified RNA was subjected to reverse transcription and qPCR for target RNA detection.

**Western blotting (WB)**

The membrane and cytoplasm were isolated according to the manufacturer's protocol (Membrane and Cytosol Protein Extraction Kit; Beyotime, Nanjing, Jiangsu, China). The isolated fractions, such as the membrane or cytoplasm, were subjected to WB blotting. In WB, protein samples were first obtained in RIPA lysis buffer (Solarbio, Beijing, China) or isolated. Proteins were separated by 12.5% sodium dodecyl sulfate-polyacrylamide gel electrophoresis and transferred to nitrocellulose (NC) membranes. It was blocked with 5% non-fat milk in TBST buffer for 1 h at room temperature and then incubated with primary antibodies overnight at 4°C. The primary antibodies used are listed in the Supplementary Table (Tab. S5). After washing, the membranes were incubated with fluorescence-conjugated secondary antibodies (IRDye® 680RD Goat anti-Mouse IgG Secondary Antibody for mouse primary antibodies, IRDye® 800CW Goat anti-Rabbit IgG Secondary Antibody for rabbit primary antibodies, LI-COR Biosciences, Lincoln, Nebraska, USA) for 1 h at room temperature. Protein bands were visualized using the Odyssey® CLx detection system (LI-COR Biosciences, Lincoln, Nebraska, USA) and quantified using analysis softwares including Image Studio (LI-COR Biosciences, Lincoln, Nebraska, USA) and ImageJ (National Institutes of Health, USA).

**ELISA and CCK-8 assay**

The ELISA assays were used to measure the cytokines in the supernatant secreted from cells, according to the manufacturer's protocol, including Human Perforin ELISA Kit (RK00135, ABclonal, Wuhan, Hubei, China), Human Granzyme B ELISA Kit (KE00121, Chicago, Illinois, USA), Human IFN-γ ELISA KIT (EH008, ExCell, Suzhou, Jiangsu, China), Human TNF-α ELISA KIT (EH009, ExCell, Suzhou, Jiangsu, China), Human TNF-β ELISA KIT (SEKH-0048, Solarbio, Beijing, China).

CCK-8 assays were performed to evaluate tumor cell proliferation according to the manufacturer's protocol (Cell Counting Kit-8, Dojindo, Kumamoto, Japan). Briefly, 100 μl of cell suspension was added to each well of a 96-well plate. Then, into each well was added 10 μl of CCK-8 solution, cultured in the incubator for 1 h, and the absorbance measured at 450 nm using a microplate reader (BioTek Synergy H1, Agilent, CA, USA).

**In vitro kinase assays**

In vitro kinase activity assay was performed using an in vitro kinase assay kit (ADP-Glo™ Kinase Assay, Promega, Madison, Wisconsin, USA) and purified proteins in accordance with the manufacturer's instructions. A 10 μl kinase reaction system was constructed in a 384-well plate, containing active purified TOPK protein (Recombinant Human PBK/TOPK Protein Active, Sino Biological, Beijing, China), 2 μg purified substrate eIF4A1 protein (Recombinant Human Eukaryotic initiation factor 4A-I, CUSABIO, Wuhan, Hubei, China), 1 μM ATP, and 1× kinase reaction buffer; meanwhile, a blank control (without TOPK and eIF4A1, only buffer and ATP) and a negative control (without TOPK, containing eIF4A1, buffer, and ATP) were set up. After the reaction was completed, an equal volume of ADP-Glo™ Reagent to the kinase reaction system was added, and incubated at room temperature for 60 minutes to terminate the reaction and deplete residual ATP; subsequently, 10 μl Kinase Detection Reagent was added, and incubated at room temperature for 30 minutes to convert ADP generated in the reaction into ATP; finally, relative light unit (RLU) values were recorded using a luminescence detector, and ADP production was calculated with reference to the standard curve to verify the phosphorylated regulatory effect of TOPK on eIF4A1.

**Truncated mutations, co-immunoprecipitation (co-IP) and mass spectrometry (MS) assay**

Truncated mutations were generated using domain-directed mutagenesis with reference to the UniProt database (https://www.uniprot.org/). PCR was performed using the wild-type plasmid DNA as a template and specific mutagenic primers. Mutated plasmids were isolated and verified by DNA sequencing. Subsequently, truncated mutants were expressed and purified. The TOPK protein (UniProtKB Entry: UQ96KB5) was subjected to truncation mutations targeting its domains, including N-terminal (1~31 amino acid, AA), kinase region 1 (31~120 AA) and 2 (120~275 AA), and C-terminal (275~322 AA) respectively, and fluorescent signals of mCherry and molecular tag of 3×Flag were labeled at the C-terminal. The eIF4A1 protein (UniProtKB Entry: P60842) was subjected to truncation mutations targeting its domains, including the N-terminal (1–31 AA), Q motif (31–62 AA), ATP-binding (62–235 AA), and P-loop (235–406 AA). Fluorescent signals of EGFP and the molecular tag of HA were labeled at the C-terminal (Fig. 3d).

Co-IP assays were performed to explore potential interacting proteins following a previously described protocol ^3^. Briefly, whole-cell lysates were prepared using IP buffer composed of 20 mM Tris-HCl (pH = 7.5), 150 mM NaCl, 10% glycerol, 1 mM EDTA, 1% TritonX, 1 mM phenylmethylsulfonyl fluoride (PMSF), 1mM Dithiothreitol (DTT), followed by centrifugation at 12,000 rpm at 4 °C for 15 min. Subsequently, the supernatants were collected and incubated with targeted antibody or IgG control at 4 °C for 2 h, followed by overnight incubation with protein A/G agarose beads (Merck, Darmstadt, Germany) at 4 °C on a rotating wheel. The beads were then washed, and the immunoprecipitated proteins were eluted and subjected to WB analysis or mass spectrometry. In addition, ANTI-FLAG magnetic beads (Millipore, Billerica, Massachusetts, USA) and DynaMag-Spin (Invitrogen, Carlsbad, California, USA) were used for the immunoprecipitation of proteins with a Flag-tag. In the MS assays, electrophoresis and silver staining of immunoprecipitated proteins were performed according to the manufacturer's protocol (Fast Silver Stain Kit; Beyotime, Nanjing, Jiangsu, China), and the targeted gels were cut for MS.

**Flow cytometry experiments**

AccuriC6 (BD Biosciences) was used for the flow cytometry assays. Data were analyzed using Modifit (Verity Software House, Topsham, Maryland, USA) and FlowJo (BD, San Jose, California, USA) to determine the cell cycle, apoptotic percentage, and protein expression intensity of the cells. In the cell cycle assay, harvested cells were washed and fixed with 70% ethanol at 4°C overnight, washed again with PBS, and stained with propidium iodide (PI) staining buffer containing RNase A for 30 min at room temperature in the dark. Flow cytometry analysis was conducted to determine the DNA content and cell cycle distribution. The apoptosis assay was performed using flow cytometry according to the manufacturer's protocol (Annexin V, FITC Apoptosis Detection Kit, Dojindo, Kumamoto, Japan). The cells were harvested and washed with ice-cold PBS, followed by resuspension in binding buffer. Annexin V-FITC and PI were added to the cell suspension and the mixture was incubated in the dark for 15 min at room temperature. After incubation, apoptotic cells were analyzed immediately, and a cell staining assay was performed to measure the expression intensity and percentage of membrane proteins such as PD-L1. The fluorescein-conjugated primary antibodies are shown in the supplementary material (Tab. S5). The obtained cells were incubated with a fluorescein-conjugated primary antibody against membrane proteins such as PD-L1 for 15 min on ice. After incubation, washing, resuspension, and cytometry assays were performed to immediately determine expression levels. An isotype control of the antibody was performed.

Multicolor flow cytometry was used to analyze cellular composition and surface protein expression in the immune microenvironment of mouse tumor tissues. Tumors were stored in MACS Tissue Storage Solution (Miltenyi Biotec, Bergisch Gladbach, Westphalia, Germany), excised from mice, minced into small pieces, and digested using a Tumor Dissociation Kit (Miltenyi Biotec, Bergisch Gladbach, Westphalia, Germany) according to the manufacturer's instructions. The digested tissues were then filtered through a 70 μm cell strainer to obtain single-cell suspensions. After washing with PBS, cells were stained with a panel of fluorochrome-conjugated antibodies specific for immune cell markers. The antibodies are listed in the supplemental materials (Tab. S5), and the gating strategy was as described by Faget et al. ^6^. Flow cytometry was conducted using a BD FACSCelesta system (BD Biosciences, San Jose, California, US). In this study, the four panels and gating strategies focused on macrophages, MDSC, DC, and lymphocytes (Fig. S8e). Data were analyzed using FlowJo software (BD Biosciences, San Jose, California, USA) to determine the phenotype and frequency of various immune cell subsets within the tumor microenvironment.

**Fluorescence-Activated Cell Sorting**

Based on the previous multicolor flow cytometry experimental procedures, BD FACSAria™ Fusion Flow Cytometers (BD, San Jose, California, USA) were used for subsequent sorting experiments. After completing voltage adjustment, channel compensation, and gating logic setting, target cells were sorted (quantity ≥ 100,000). They were collected by centrifugation, resuspended in an appropriate cell culture medium, stabilized in a 37°C cell incubator, followed by cell counting and viability detection.

**Seahorse Mito stress assay**

Seahorse XF cell Mito stress test was performed in accordance with the manufacturer's instructions (Seahorse XF Mito Stress Test Kits, Agilent, Santa Clara, California, USA). Specifically, Target active T cells were isolated by flow cytometry sorting, resuspended in cell assay medium (Seahorse XF RPMI Medium pH 7.4, Agilent, Santa Clara, California, USA) with glucose, pyruvate and glutamine solution (Agilent, Santa Clara, California, USA), and stabilized in vitro. The cell suspension was seeded into Seahorse cell culture plates pre-coated with Poly-D-lysine (Sangon Biotech, Shanghai, China) and pre-warmed overnight in a 37°C CO₂-free incubator, with 50 μL of suspension containing 100,000 cells per well. Five biological replicates were set for each experimental condition, and background control wells without cells were set at the four corners of the cell plate with 50 μL of assay medium added. Cells were centrifuged at 200×g for 1 minute to attach the suspended cells to the bottom of the wells. The pre-hydrated sensor cartridge (Seahorse XFe96/XF Pro FluxPak Mini, Agilent, Santa Clara, California, USA) loaded with drugs was combined with the cell plate and placed into the Seahorse XFe96 Analyzer (Agilent, Santa Clara, California, USA), with 25 μL of compound solution added to each injection port. According to the preset program, the instrument automatically injected Oligomycin (1.5 μM), Carbonyl cyanide-4 (trifluoromethoxy) phenylhydrazone (FCCP, 1 μM), and Rotenone/Antimycin A mixture (R/A, 0.5 μM) sequentially. Each condition was cyclically detected 3 times, each including mixing, waiting, and reading steps. The oxygen consumption rate (OCR) of each well was recorded and calculated in real-time by the instrument. Based on the OCR kinetic curves generated by the instrument, the basal OCR (stable OCR value before compound injection) and spare capacity (difference between maximum respiratory rate and basal respiratory rate) of the cells were calculated, and statistical methods were used for intergroup difference comparison.

**Proteomics and phosphoproteomics assay**

The cell samples were sonicated three times on ice using a high-intensity ultrasonic processor in a lysis buffer containing a 1% protease inhibitor cocktail. The supernatant was collected, and the protein concentration was determined using a BCA kit (Pierce BCA Protein Assay Kit, Thermo Fisher Scientific, Massachusetts, USA). The protein was then digested into peptides, and affinity enrichment was performed for phosphopeptides. The peptides were subjected to a 4D LC-MS/MS assay, and the resulting data were processed using the MaxQuant search engine (v.1.6.15.0). The analysis utilized the "Homo_sapiens_9606_SP_20230103. The FASTA " database contains 20,389 protein sequences. Trypsin/P was designated as enzyme cleavage-specific, allowing for up to two missed cleavage sites. The accuracy of the identification of the spectra, peptides, and proteins was set at 1% FDR. The relative quantification values of the ratio of phosphorylated proteins to total proteins were used for downstream differential expression analysis of post-translational modification. When *P* < 0.05, an absolute value of fold change exceeding 1.5 is used as the significance threshold for differential expression. GO analysis was performed to identify the enriched pathways of the differentially expressed proteins. For motif analysis, the MoMo analysis tool based on the motif-x algorithm was used to analyze the motif characteristics of modification sites ^7^. Based on the MoMo analysis, the degree of change in the frequency of amino acids near the modification sites was scored and presented as a heat map. Based on the fold change in phosphorylated proteins, they were further divided into four sections, referred to as Q1 to Q4, accounting for one-quarter of the total population in each Q group. The Pfam database (https://pfam.xfam.org/) was used for the protein domain enrichment analysis. Fisher's exact test was used to assess the significance of enrichment among differentially expressed proteins. Statistical significance was defined as *P* < 0.05.

**Metabolomics**

The samples were thawed, rinsed, crushed, mixed with 70% methanol, vortexed, and centrifuged. The supernatant was transferred to another metabolome analysis. The acquisition of tryptophan and its metabolites contents were performed using Ultra Performance Liquid Chromatography (UPLC) by ExionLC™ AD system and Tandem Mass Spectrometry (MS/MS) by QTRAP® 6500+ (SCIEX, Fresno, California, USA). Thirty-one metabolites in the tryptophan-kynurenine pathway were included for detection (Metware Database, Wuhan, Hubei, China). The data were processed using Analyst 1.6.3 software and MultiQuant 3.0.3 software (SCIEX, Fresno, California, USA). The retention times and peak information of reference standards were referenced to perform peak integration correction for chromatographic peaks detected in different samples, ensuring the accuracy of qualitative and quantitative analysis. The quantitative calculation is as follows: (The content of amino acid in solid samples, ng/g) = (The concentration values obtained by substituting the peak area of the sample into the standard curve, ng/ml) × (The volume of the extraction solution used during extraction, μl) / (The mass of the sample, g) / 1000. OPLS-DA was used to decompose the X-matrix information into two types, related and unrelated to Y, by removing the unrelated differences to filter the differential variables. The data were log_2_ transformed and mean-centered, and OPLS-DA was performed using the OPLSR.Anal function in the MetaboAnalystR package (Version 1.0.1) within the R software. Significantly regulated metabolites between groups were determined using Variable Importance in Projection (VIP) and absolute Log_2_FC.

**Molecular docking**

In the present study, molecular docking was performed to investigate the interactions between TOPK inhibitors and TOPK. The three-dimensional structure of the protein was obtained from the PDB database (https://www.rcsb.org/), and compounds such as OTS964 and OTS514 were obtained from PubChem (https://pubchem.ncbi.nlm.nih.gov/). Docking simulations were performed, and the binding affinity of the ligand to the receptor was evaluated based on the calculated docking score or binding energy. The results of the molecular docking provided insights into the potential binding modes and key interactions between the compound and target. A binding energy under -7.0 kcal/mol was considered a strong binding affinity and stabilization between the compound and the target. Protein docking of TOPK and eIF4A1 was performed based on the results of truncated mutations. The three-dimensional structures of the proteins were obtained from the AlphaFold Protein Structure Database (https://alphafold. ebi. ac=/). Rigid docking simulations were performed using Global Range Molecular Matching (GRAMM) docking software (http://vakser.compbio.ku.edu/resources/gramm/) to predict the interactions between the AlphaFold-derived predicted proteins, which were consistent with the co-IP results of truncated mutations. Docking visualization was performed using the PyMol software (Version 2.1, https://pymol.org/).

**Ribo-seq assay and ribosome profiling**

Ribo-seq is the mainstream translatomics method. The specific procedure involves treating ribosome-nascent peptide complexes with low RNase concentrations to degrade RNA fragments not shielded by ribosomes, followed by ribosome removal. Next-generation sequencing was used to detect short RNA fragments protected by actively translating ribosomes. These RPFs accurately represent the footprints of the translation in progress. First, adherent cells were incubated in a fresh DMEM medium containing 0.1 mg/ml Cycloheximide (CHX) for 1 min, then washed with pre-chilled PBS containing 0.1 mg/ml CHX. Cell suspensions were collected using a cell scraper, and RPFs were isolated following a previously described protocol ^8^. Simultaneous transcriptional profiling was conducted alongside Ribo-seq library preparation. A nine-quadrant diagram was used to visualize the results of the integrated analysis of the translatome and transcriptome. Proteomaps were used to visualize enriched RPFs ^9^. Translated upstream open reading frame (uORF) is believed to regulate the TE of mRNA translation. Ribosome profiling assays were performed to validate mRNA distribution profiles in polysome fractionation, following established protocols ^10,11^.

**Bioinformatics**

The scRNAs of gastric cancer were analyzed following the data and methods outlined by Lee et al. ^12^. The transcriptomic expression profiles and corresponding clinical information for gastric cancer were retrieved from the TCGA dataset (https://portal.gdc.com). Differentially expressed mRNAs were investigated using the limma package in R software, with thresholds defined as adjusted *P* < 0.05 and |Log2 (FC)| > 1." A functional enrichment analysis was conducted to elucidate the potential functions of the identified targets. Gene Ontology serves as a fundamental tool for gene annotation encompassing biological pathways, molecular functions, and cellular components. KEGG enrichment analysis provides valuable insights into gene functions and associated high-level genome functional information. The ClusterProfiler package (version 3.18.0) was used for Gene Ontology (GO) analysis and enrichment of Kyoto Encyclopedia of Genes and Genomes (KEGG) pathways. Visualization was performed using the ggplot2 package in R software (version 4.3.1). The GSVA package was employed for analysis, with the parameter set to 'ssgsea' method and reference to MsigDB (https://www.gsea-msigdb.org/), and the correlation between genes and pathway scores was assessed using Spearman correlation analysis in R (Version 4.0.3). *P* value <0.05 was considered statistically significant.

**Statistics**

Statistical analysis was performed utilizing GraphPad Prism 9 or SPSS 25. *P* values were determined using a two-tailed Student's t-test for comparison between two groups or ANOVA for comparison between more than two groups. Survival curves were constructed using the Kaplan–Meier method and subsequently compared using the log-rank test. Experiments were independently conducted three times in vitro and five times in vivo. Error bars represent the mean ± SD of replicates. Statistical significance was determined using an unpaired two-tailed Student's t-test (**P* < 0.05, ***P* < 0.01, ****P* < 0.001).

**Supplemental Figure Legend**

**Supplemental Fig. S1: Integrated CRISPR and compounds screening identifies inhibitors blocking IFN-γ-induced PD-L1 (Fig. 1 supplement)**

**a** Kyoto Encyclopedia of Genes and Genomes (KEGG) pathway annotation of the mediators in IFN-γ induced PD-L1 pathway in gastric cancer cells. **b** The selected kinases with specific inhibitors annotated in the human protein kinome dendrogram. **c** Flowchart of the compound screening of selected kinase inhibitors interfering with the cytotoxicity of T cells from peripheral blood mononuclear cells (PBMC-T, 3 donors) against gastric cancer cells. **d** Real-time monitoring model for the co-culture of labeled live cells in the Incucyte system. The bright and dark phases of the model are also shown. **e** Granzyme B secreted from T cells from peripheral blood mononuclear cells (PBMC-T) co-cultured with gastric cancer cells in the presence of an inhibitor for 48h. Inhibition with > 1000 pg/ml granzyme B was selected. Experiments were independently performed at least three times in vitro. **f** Molecular docking model and binding measurements of compounds (OTS964 and OTS514) and TOPK. **g** TOPK protein levels determined by western blotting in gastric cancer cell lines. **h** Upregulation or downregulation of TOPK protein in gastric cancer cells (SGC7901, BGC823, and NCI-N87) using western blotting. All experiments were performed independently at least three times in vitro. Error bars represent the mean±SD of independent replicates. *P* values were determined using unpaired two-tailed Student's t-tests.

**Supplemental Fig. S2: Protein kinase TOPK inhibitors efficiently suppresses gastric cancer proliferation (Fig. 1 supplement)**

**a** Growth inhibition curves of dose and time gradients in cell lines (SGC7901, BGC823, NCI-N87, MGC803, MFC, and CT26) treated with OTS964. **b** Growth inhibitory curves of dose- and time-gradients in cell lines (BGC823, NCI-N87, and MFC) treated with OTS514. **c** OTS964 (25 and 50 nM) effectively inhibited colony formation in gastric cancer cells. **d** OTS964 (50 nM) effectively inhibited colony formation of SGC7901 over a time gradient (24h, 48h, 72h). **e** OTS964 effectively induced apoptosis in gastric cancer cells at dose gradient (25, 50, and 100 nM). **f** OTS964 induces G2/M cell cycle arrest in gastric cancer cells at dose gradients (25, 50, and 100 nM). **g** OTS964 disrupts the mitochondrial membrane potential, leading to an increase in JC-1 monomers. **h** OTS964 and OTS514 effectively inhibited tumor spheroid formation and 3D viability of gastric cancer cells (SGC7901, BGC823, NCI-N87, and MFC) at IC_50_ dose at 72h. **i** OTS964 inhibits the growth of subcutaneous xenograft cells in vivo in BALB/c nude mice. All experiments were performed independently at least three times in vitro and five times in vivo. Error bars represent the mean±SD of independent replicates. *P* values were determined using unpaired two-tailed Student's t-tests. **P* < 0.05, ***P*  < 0.01, ****P*  < 0.001.

**Supplemental Fig. S3: Bioinformatics analysis of expression and phosphorylated function of protein kinase TOPK in gastric cancer (Fig. 2 supplement)**

**a** High TOPK expression in tumors in the pan-cancer database (The Cancer Genome Atlas, TCGA). **b** High TOPK expression in gastric cancer data (Asian Cancer Research Group, ACRG; Peking University Cancer Hospital, PUCH). **c** Gene Ontology (GO) and Kyoto Encyclopedia of Genes and Genomes (KEGG) pathway annotation of upregulated differentiated genes. Patients in the top 25% with high TOPK expression were compared with those in the top 25% with low expression in Stomach Adenocarcinoma (STAD) data (n=188). Colors denote the significance of divergent enrichment, whereas the dimensions of the circles reflect the gene count, with larger circles indicating a greater number of genes. **d** TOPK and MKI67 expression analysis and distribution in the scRNA data of gastric cancer. **e** Spearman correlation analysis of TOPK expression and ssGSEA pathways in STAD data (n=375). The density curve on the right illustrates the distribution trend of pathway scores, whereas the upper-density curve delineates the trend in gene expression distribution. **f, g** Volcano plot and Gene Ontology (GO) pathway annotation of differentially expressed proteins (**f**) and phosphorylated proteins with phosphorylation sites (**g**) in gastric cancer cells showing differential TOPK expression. n=3 replicates. Red dots indicate upregulated genes, blue dots indicate downregulated genes and gray dots indicate non-significance. **c** Serine and threonine motif analysis of TOPK in gastric cancer cells. **h** Serine and threonine motif analysis of TOPK in gastric cancer cells. **i** Differentially structured domains of proteins influenced by TOPK phosphorylation.

**Supplemental Fig. S4: Protein kinase TOPK promotes proliferation, invasion, and metastasis of gastric cancer (Fig. 2 supplement)**

**a** TOPK knockdown or overexpression influences gastric cancer cell proliferation monitored via Incucyte system. **b, c** TOPK expression influences tumor spheroid formation and 3D viability of gastric cancer cells SGC7901, BGC823 (**b**) and MFC (**c**) at 72h. **d** Regulation of TOPK expression showed cell proliferation in EdU and apoptosis in the TUNEL assay. **e-f** TOPK expression influences colony formation (**e**), wound healing (**f**), migration, and invasion (**g**) of gastric cancer cells. **h** TOPK expression influences the growth of subcutaneous cell xenografts in vivo in BALB/c nude mice (n=5). **i** TOPK expression influences lung metastasis and growth of gastric cancer cells in vivo in BALB/c nude mice (n=6 replicates). In the fluorescence intensity plot, if the lower limit of the error bar extends beyond the axis range, the segment below indicates that the error is not displayed. Experiments were performed independently at least three times in vitro and five times in vivo, and one representative image is shown. Error bars represent the mean±SD of independent replicates. *P* values were determined using unpaired two-tailed Student's t-tests. **P* < 0.05, ***P*  < 0.01, ****P*  < 0.001.

**Supplemental Fig. S5: TOPK mediates IFN-γ induced PD-L1 and IDO1 expression (Fig. 2 supplement)**

**a** Higher TOPK expression in response patients of immunotherapy cohorts and ROC curve (Pender et al., IMvigor210, Rose et al.). **b** Correlation between TOPK expression and immune cell infiltration (Treg, Tex, Tfh, NKT, NK, and MAIT cells) in the TCGA-STAD and ACRG datasets. **c** IFN-γ at dose gradients induced overexpression of proteins including STAT1, PD-L1, IDO1, and phosphorylated proteins including p-TOPK-Tyr74 and p-STAT1-Tyr701 in SGC7901 for 48h. **d** IFN-γ at dose gradients induced overexpression of membrane PD-L1 in SGC7901 and BGC823 for 48h. The dashed line represents the median fluorescence intensity (MFI) of the mock group. **e** TOPK knockdown inhibited CCK-8 cell viability but not IFN-γ in gastric cancer cells. **f** IFN-γ secretion under different effector-to-target ratios of PBMC-T to BGC823 cell at 48h. **g** TOPK overexpression decreased PBMC-T cell cytotoxicity against gastric cancer cells for 48h. **h** Knockdown of PD-L1 or IDO1 expression in gastric cancer cells reversed the inhibitory effect of TOPK overexpression on the secretion of GZMB and Perforin by PBMC-T cells. Gastric cancer cells were treated with 10ng/ml IFN-γ for 48h to simulate the IFN-γ condition. Experiments were performed independently at least three times in vitro, and one representative image is shown. Error bars represent the mean±SD of independent replicates. *P* values were determined using unpaired two-tailed Student's t-tests. **P* < 0.05, ***P*  < 0.01, ****P*  < 0.001.

**Supplemental Fig. S6: TOPK enhances *STAT1* mRNA translation via phosphorylating eIF4F complex components (Fig. 3 supplement)**

**a** Score and rank of predicted TOPK phosphorylation sites of eIF4A and eIF4F complex components based on the atlas of substrate specificities for the human kinome. **b** Predicted phosphorylation interaction network of TOPK and eIF4F complex components. **c** Western blot assays showing eIF4A1 knockdown in gastric cancer cells (SGC7901 and BGC823). **d, e** eIF4A1 knockdown blocks IFN-γ induced membrane PD-L1 (**d**) and total STAT1, PD-L1 and IDO1 (**e**) overexpression in gastric cancer cells. The dashed line represents the median fluorescence intensity (MFI) in the negative control (NC) group with IFN-γ. **f, g** Intracellular co-localization of TOPK, eIF4E (**f**), and eIF4B (**g**) in gastric cancer cells. **h** Differentially expressed ribosome-protected fragments (RPFs) via Ribo-seq in SGC7901 cells with TOPK knockdown compared with the control. **i** Proteomap of downregulated RPFs (dRPFs) and transcription factors with downregulated translation efficiency (TE) in dRPFs. **j** Open reading frame of *STAT1* mRNA would not be detected in RPFs after TOPK knockdown in SGC7901 under IFN-γ condition. **k** Ribosome profiling assays reveal TOPK knockdown would not influence mRNAs of *GAPDH*, *eIF4A1*, *eIF4E*, *STAT3*, and *JAK2* in ribosome polysomes under IFN-γ condition. **l** Diagram of the IFN-γ-TOPK-eIF4F-STAT1-PD-L1/IDO1 axis in gastric cancer. Gastric cancer cells were treated with 10ng/ml IFN-γ for 48h to simulate IFN-γ condition. Experiments were independently performed at least three times in vitro, and representative images are shown. Error bars represent the mean±SD of independent replicates. *P* values were determined using unpaired two-tailed Student's t-tests. **P* < 0.05, ***P*  < 0.01, ****P*  < 0.001.

**Supplemental Fig. S7:** **Phosphorylation of the key sites of eIF4A1 targeted by TOPK affects the translation efficiency of *STAT1***  **(Fig. 4 supplement)**

**a** The inactive mutations S78A and T158A of eIF4A1 inhibit the ribosomal translation efficiency of *STAT1* mRNA. **b** The inactive mutations S78A and T158A of eIF4A1 inhibit the gene transcriptional expression of *PD-L1* and *IDO1*, but do not affect *STAT1*. **c** The inactive mutations S78A and T158A of eIF4A1 inhibit the protein expression of STAT1, PD-L1, and IDO1. **d** The activating mutations S78E and T158E of eIF4A1 enhance the translation efficiency of *STAT1* and can reverse the decreased ribosomal translation efficiency of *STAT1* mRNA induced by TOPK inhibition; they have no significant effect on *PD-L1* and *IDO1* mRNA. **e** The activating mutations S78E and T158E of eIF4A1 have no significant effect on *STAT1* mRNA, but exert a notable impact on the gene transcriptional expression of *PD-L1* and *IDO1*, the downstream target genes of STAT1 protein. **f** The activating mutations S78E and T158E of eIF4A1 exert a significant effect on the protein expression of STAT1, PD-L1, and IDO1. **g** The inactive mutation S394A and activating mutation S394E of eIF4A1 have no significant effect on the ribosomal translation efficiency of *STAT1*, *PD-L1*, and *IDO1* mRNA. **h** The inactive mutation S394A and activating mutation S394E of eIF4A1 have no significant effect on the mRNA expression of STAT1, PD-L1, and IDO1. **i** The inactive mutation S78A of eIF4A1 inhibits its binding to *STAT1* mRNA, while the activating mutation S78E promotes this binding. **j** The inactive mutation T158A of eIF4A1 inhibits its binding to *STAT1* mRNA, while the activating mutation T158E promotes this binding. **k** Neither the inactive mutation S394A nor the activating mutation S394E of eIF4A1 has an effect on its binding to *STAT1* mRNA. Cancer cells were treated with 50 nM OTS964 for 48h. Gastric cancer cells were treated with 10ng/ml IFN-γ for 48h to simulate IFN-γ condition. Experiments were independently performed at least three times in vitro, and representative images are shown. Error bars represent the mean±SD of independent replicates. *P* values were determined using unpaired two-tailed Student's t-tests. **P* < 0.05, ***P*  < 0.01, ****P*  < 0.001.

**Supplemental Fig. S8: Phosphorylation of the key sites of eIF4B and eIF4E targeted by TOPK exerts a weak regulatory effect on the translation efficiency of *PD-L1* and *IDO1* (Fig. 4 supplement)**

**a** The inactive mutations S422A of eIF4B and S209A of eIF4E cause a weak decrease in the ribosomal translation efficiency of P*D-L1* and *IDO1* mRNA. **b** The active mutations S422E of eIF4B and S209E of eIF4E cause a weak increase in the ribosomal translation efficiency of P*D-L1* and *IDO1* mRNA. **c, d** The activating mutation S422E of eIF4B (**c**) or S209E of eIF4E (**d**) fails to reverse the decreased ribosomal translation efficiency of *STAT1* mRNA induced by eIF4A1 inhibition. **e** RNA binding protein immunoprecipitation (RIP)-qPCR assay analysis showed that neither the inactive nor activating mutations of eIF4B-S422 and eIF4E-S209 affect the binding of eIF4A1 to *STAT1* mRNA. Cancer cells were treated with 50 nM OTS964 for 48h. Gastric cancer cells were treated with 10ng/ml IFN-γ for 48h to simulate IFN-γ condition. Experiments were independently performed at least three times in vitro, and representative images are shown. Error bars represent the mean±SD of independent replicates. *P* values were determined using unpaired two-tailed Student's t-tests. **P* < 0.05, ***P*  < 0.01, ****P*  < 0.001.

**Supplemental Fig. S9: TOPK inhibitor synergizes with immune checkpoint blockade for gastric cancer therapy (Fig. 6 supplement)**

**a** Real-time monitoring model excluding PBMC-T cells demonstrates that anti-CTLA-4 or anti-PD-1 antibodies exhibit no additional effect on gastric cancer cell proliferation. **b, c** OTS964 combined with anti-PD-1 and anti-CTLA-4 antibodies (**b**) or dual anti-PD-1/CTLA-4 antibody (cadonilimab) (**c**) exhibits synergistic effects in increasing T cell cytotoxicity against gastric cancer cells. anti-CTLA-4 antibody or isotype, 100 nM; anti-PD-1 antibody or isotype, 20 nM; dual anti-PD-1/CTLA-4 antibody or isotype, 50 nM. **d** OTS964, or combined with anti-CTLA-4 or anti-PD-1 antibody, enhances TNF-β secretion rather than TNF-α from PBMC-T cells in a co-culture model targeting gastric cancer cells. anti-CTLA-4 antibody or isotype, 100 nM; and anti-PD-1 antibody or isotype, 20 nM. **e** multi-color flow cytometry assay panels and gating. Gastric cancer cells were treated with 10ng/ml IFN-γ for 48h to simulate IFN-γ condition. **f** Multi-color flow cytometry assays revealed that TOPK knockdown increased immune cell infiltration, including CD4+ or CD8+ T cells, NK and NKT cells, cDC cells, and the M1/M2 ratio, and CD25+ Tregs or myeloid-derived suppressor cells (MDSC) were reduced in 615 mice model with MFC-derived allograft. Experiments were independently performed three times in vitro, and representative images are shown. Error bars represent the mean±SD of independent replicates. *P* values were determined using unpaired two-tailed Student's t-tests. **P* < 0.05, ***P*  < 0.01, ****P*  < 0.001.

**Supplemental Fig. S10: TOPK is associated with clinical gastric cancer malignant phenotype (Fig. 7 supplement)**

**a** Immunohistochemical staining for TOPK in normal, adjacent, and tumor tissues from clinically verified patients with gastric cancer. **b** Representative images of low or high immunohistochemical scores of TOPK in the cytosol of tumor samples. **c** Patients responding to immunotherapy for gastric cancer have a higher probability of high cytoplasmic TOPK expression in tumor cells before treatment. **d** Patients with high TOPK expression have a poorer prognosis for survival in the pan-cancer database. **P* < 0.05. **e** Patients with high cytoplasmic TOPK expression in tumor cells have a higher percentage and density of αSMA⁺ CAFs and CD163⁺ M2 macrophages in the tumor microenvironment. **f, g** Illustration (**f**) and comparison of proximity ligation assays (PLA) of the spatial proximity interaction of TOPK and eIF4A1 in adjacent and tumor tissues of patients with gastric cancer (**g**). TOPK was conjugated with a green fluorescent dye labeled CoraLitePlus-488 to visualize the spatial sublocalization of the TOPK protein in situ in cells. **h** Comparison of proximity ligation assays (PLA) of the spatial proximity interaction of TOPK and eIF4B or eIF4E proteins in adjacent and tumor tissues of gastric cancer patients. **i** Simplified mechanism schematic diagram of TOPK activating eIF4A1 to regulate the ribosome-protected fragments (RPF) and mRNA of the related targets such as PD-L1 and IDO1 in the IFN-γ/STAT1 pathway. **j** Diagram of the TOPK inhibitors OTS964 or OTS514 improving the immune microenvironment in gastric cancer tumors. Experiments were performed independently at least three times in vitro. Error bars represent the mean±SD of independent replicates. *P* values were determined using unpaired two-tailed Student's t-tests. **P* < 0.05, ***P*  < 0.01, ****P*  < 0.001.

**Supplemental Table Legend**

Table S1. The potential compounds screened out by meeting the threshold.

Table S2. The downregulated ribosome-protected fragments (dRPF) with downregulated translation efficiency (TE) in transcription factors (TF) and value.

Table S3. The regulatory influence of TOPK on STAT1 RPFs and open-read fragments (ORF) type.

Table S4. The IHC scores of TOPK in the nucleus, cytoplasm, and total tumor cells of gastric cancer patient samples.

Table S5. Antibodies purchased in this study, along with their sources and identifiers.

Table S6. Chemicals, peptides, and recombinant proteins purchased in this study, along with their sources and identifiers.

Table S7. Critical commercial kits purchased in this study, along with their sources and identifiers.

Table S8. Nucleotides used in this study, along with their sources and sequences.

Table S9. Plasmids and nucleotides used in this study, along with their sources and elements.

**REFERENCE FOR SUPPLEMENTAL MATERIALS AND METHODS**

1 Tentler, J. J. *et al.* Patient-derived tumour xenografts as models for oncology drug development. *Nat Rev Clin Oncol* **9**, 338-350, doi:10.1038/nrclinonc.2012.61 (2012).

2 Deng, Z. *et al.* Transcriptome profiling of patient-derived tumor xenografts suggests novel extracellular matrix-related signatures for gastric cancer prognosis prediction. *J Transl Med* **21**, 638, doi:10.1186/s12967-023-04473-0 (2023).

3 Huangfu, L. *et al.* Piceatannol enhances Beclin-1 activity to suppress tumor progression and its combination therapy strategy with everolimus in gastric cancer. *Sci China Life Sci* **66**, 298-312, doi:10.1007/s11427-022-2185-9 (2023).

4 Han, J. *et al.* SDCBP-AS1 destabilizes beta-catenin by regulating ubiquitination and SUMOylation of hnRNP K to suppress gastric tumorigenicity and metastasis. *Cancer Commun (Lond)* **42**, 1141-1161, doi:10.1002/cac2.12367 (2022).

5 Chen, J. *et al.* Death receptor 5 promotes tumor progression in gastric cancer. *FEBS Open Bio* **13**, 2375-2388, doi:10.1002/2211-5463.13725 (2023).

6 Faget, J. *et al.* Neutrophils and Snail Orchestrate the Establishment of a Pro-tumor Microenvironment in Lung Cancer. *Cell Rep* **21**, 3190-3204, doi:10.1016/j.celrep.2017.11.052 (2017).

7 Cheng, A., Grant, C. E., Noble, W. S. & Bailey, T. L. MoMo: discovery of statistically significant post-translational modification motifs. *Bioinformatics* **35**, 2774-2782, doi:10.1093/bioinformatics/bty1058 (2019).

8 Ingolia, N. T., Brar, G. A., Rouskin, S., McGeachy, A. M. & Weissman, J. S. The ribosome profiling strategy for monitoring translation in vivo by deep sequencing of ribosome-protected mRNA fragments. *Nat Protoc* **7**, 1534-1550, doi:10.1038/nprot.2012.086 (2012).

9 Liebermeister, W. *et al.* Visual account of protein investment in cellular functions. *Proc Natl Acad Sci U S A* **111**, 8488-8493, doi:10.1073/pnas.1314810111 (2014).

10 Panda, A. C., Martindale, J. L. & Gorospe, M. Polysome Fractionation to Analyze mRNA Distribution Profiles. *Bio Protoc* **7**, doi:10.21769/BioProtoc.2126 (2017).

11 Yuan, M. *et al.* Cancer-associated fibroblasts employ NUFIP1-dependent autophagy to secrete nucleosides and support pancreatic tumor growth. *Nat Cancer* **3**, 945-960, doi:10.1038/s43018-022-00426-6 (2022).

12 Lee, S. H. *et al.* Apposition of Fibroblasts With Metaplastic Gastric Cells Promotes Dysplastic Transition. *Gastroenterology* **165**, 374-390, doi:10.1053/j.gastro.2023.04.038 (2023).
